# Fully autonomous tuning of a spin qubit

**DOI:** 10.1038/s41928-025-01562-4

**Published:** 2026-02-02

**Authors:** Jonas Schuff, Miguel J. Carballido, Madeleine Kotzagiannidis, Juan Carlos Calvo, Marco Caselli, Jacob Rawling, David L. Craig, Barnaby van Straaten, Brandon Severin, Federico Fedele, Simon Svab, Pierre Chevalier Kwon, Rafael S. Eggli, Taras Patlatiuk, Nathan Korda, Dominik M. Zumbühl, Natalia Ares

**Affiliations:** 1 Department of Materials, University of Oxford, Oxford, UK; 2 Department of Physics, University of Basel, Basel, Switzerland; 3 Mind Foundry, Oxford, UK; 4 Department of Engineering Science, University of Oxford, Oxford, UK; 5 Present Address: School of Electrical Engineering and Telecommunications, UNSW Sydney, Sydney, New South Wales Australia

**Keywords:** Electronic devices, Condensed-matter physics

## Abstract

The development of large-scale semiconductor quantum circuits is limited by the difficulties involved in efficiently tuning and operating such circuits. Identifying optimal operating conditions for these qubits is, in particular, complex and involves the exploration of vast parameter spaces. Here we report the autonomous tuning of a semiconductor qubit, from a grounded device to Rabi oscillations. Our approach integrates deep learning, Bayesian optimization and computer vision techniques. We demonstrate this automation in a germanium–silicon core–shell nanowire device. To illustrate the potential of full automation, we characterize how the Rabi frequency and *g*-factor depend on barrier gate voltages for one of the qubits found by the algorithm. We expect our automation algorithm to be applicable to a range of semiconductor qubit devices, allowing for the statistical studies of qubit-quality metrics.

## Abstract (web summary)

An algorithm that combines deep learning, Bayesian optimization and computer vision techniques can be used to autonomously tune a semiconductor spin qubit from a grounded device to Rabi oscillations.

## Main

Spin qubits in semiconductor devices could be used to build a universal quantum computer^1–11^. Recent work with such systems has demonstrated two-qubit gates with fidelities that surpass the thresholds for fault-tolerant computing^4,12,13^ and hot qubits that can address the bottleneck of millikelvin refrigeration^7,14,15^. This has been accompanied by advances in the wafer-scale manufacturing of these devices^16,17^ as well as their efficient testing at cryogenic temperatures^18,19^. However, semiconductor quantum circuits are currently limited to 12 qubits in one device^16^, despite the fact that modern semiconductor fabrication techniques could support the integration of millions of qubits.

A key reason for this limitation is the intricate tuning required to reach and maintain qubit operation. A range of approaches have been explored to automate single stages of this process, including defining double-quantum-dot (DQD) confinement potentials^20–23^, navigating to specific charge transitions^24–32^, fine tuning of charge transport features^33^ and interdot tunnel couplings^34,35^, as well as device characterization^36–40^. These works offer a glimpse into the potential of machine learning for full qubit tuning automation, but the challenge remains.

**Fig. 1:**
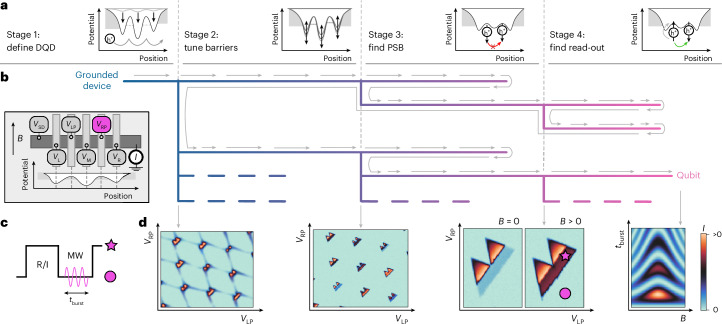
Algorithm overview. **a**, There are four stages that the algorithm needs to successfully navigate to reach qubit operations. The goal of each stage is illustrated with a confinement potential diagram. **b**, Each stage can either be successful and produce candidates (leading to one or more branches), or unsuccessful (leading to backtracking to the closest stage that still has candidates). The search is, therefore, conducted in a tree structure. Some branches of the tree might be left unexplored. This is indicated by the dashed lines. Inset: illustration of the device. Five different voltages *V*_*i*_ can be applied to a linear confinement. **c**, A pulsing scheme can be applied with an MW burst of length *t*_burst_. The fast line is connected to the plunger gate RP. **d**, Simulated measurement illustrations that mark the successful transition between stages. Starting from a stability diagram with mere DQD features (far left), the algorithm tunes the parameters until promising bias triangles (middle left), triangles exhibiting PSB (middle right) and finally Rabi oscillations (far right) are obtained. The extremal points of the pulse scheme are indicated as a star and circle in the middle-right illustration.

Here we report a fully autonomous tuning process that can encode a qubit without the need for human intervention. The process of going from a fully de-energized device to the observation of Rabi oscillations, a definitive indicator of qubit functionality, typically takes human experts weeks, or even months, to complete. Our algorithm (Fig. 1), deployed on a DQD device, can complete the tuning process within three days. Our approach integrates deep learning, Bayesian optimization and a computer vision technique, and its success lies in the ability of the algorithm to navigate through various stages of the tuning process, efficiently handling challenges and making accurate decisions.

## Device architecture and read-out technique

**Extended Data Fig. 1:**
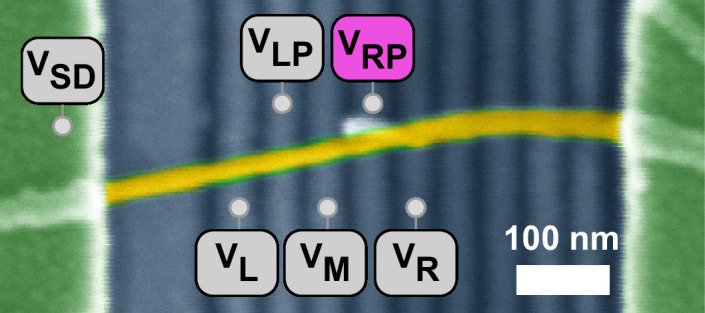
Device. False color micrograph of a nanowire device similar to the one used in this work. The Ge/Si nanowire is colored in yellow.

We use a Ge–Si core–shell nanowire device (Extended Data Fig. 1) in which holes are confined in depletion mode^41^. The electrical potential is set by a number of gate electrodes. Two plunger gate electrodes predominantly shift the electrochemical potential in the left and right dots with voltages *V*_LP_ and *V*_RP_, respectively. The remaining gate electrodes primarily control the barriers between the DQD and the leads as well as the interdot coupling. One of the plunger gates is connected to a high-frequency line via a bias-tee, allowing for the application of voltage pulses and microwave (MW) bursts.

The device can be probed by applying a bias voltage *V*_SD_ to the source lead and recording the current *I*_SD_ at the drain lead. The algorithm navigates to a DQD occupation that exhibits Pauli spin blockade (PSB) for spin-to-charge conversion. To achieve this, the DQD does not need to be depleted to the single-hole regime. The charge occupation on each dot is estimated to be in the range of several dozens^42–45^.

The algorithm uses a two-stage pulsing protocol^15,46–48^, which is parameterized by an MW pulse frequency and duration *t*_burst_ (Fig. 1c). This protocol allows for qubit manipulation if the spin resonance conditions are met. Details on the pulsing scheme for coherent spin control and the device are described in the Methods and another work^41^.

## Autonomous tuning procedure

The autonomous tuning algorithm is structured into four main stages. Starting from a completely de-energized device, that is, with all gate voltages set to 0, the first two stages define the DQD potential by tuning the interdot barrier and the reservoir coupling. The third stage narrows the search space by looking for distinct signatures of PSB, an initialization and read-out requirement. The last stage fine-tunes the plunger voltages and finds the frequency and duration of an MW pulse needed to drive the qubit. The effect of each stage on the DQD confinement potential is illustrated in Fig. 1a. Measurement illustrations exemplifying those taken by the algorithm are shown in Fig. 1d.

As a result of the algorithm design, a search tree emerges (Fig. 1b). Once a stage is successfully completed, a list of candidates is generated. A candidate consists of all the information needed for the next stage to investigate it, usually containing locations or ranges of gate voltages, or information on the suspected *g*-factor and Rabi frequency *f*_Rabi_. The candidates are ordered by a dedicated score in each stage and a single candidate is passed onto the next stage. If a stage is unsuccessful, the algorithm backtracks to the previous stage and investigates the next candidate in that stage’s list of candidates. This process dynamically creates a search tree (Supplementary Fig. 7 shows examples from real tuning runs). If a different branch has proven to lead to a qubit, some branches of the tree may be left unexplored. These are indicated by dashed lines in the tree in Fig. 1.

We describe each stage in this section. A list of all stages and substages is provided in Extended Data Table 1. More details are provided in Methods and details on the stage structure and composition of candidates for each stage are provided in Supplementary Section 8.

The first stage identifies the gate voltage settings that define the DQD confinement potential. It determines a lower and upper limit for each barrier gate voltage, which is used in subsequent stages.

**Fig. 2:**
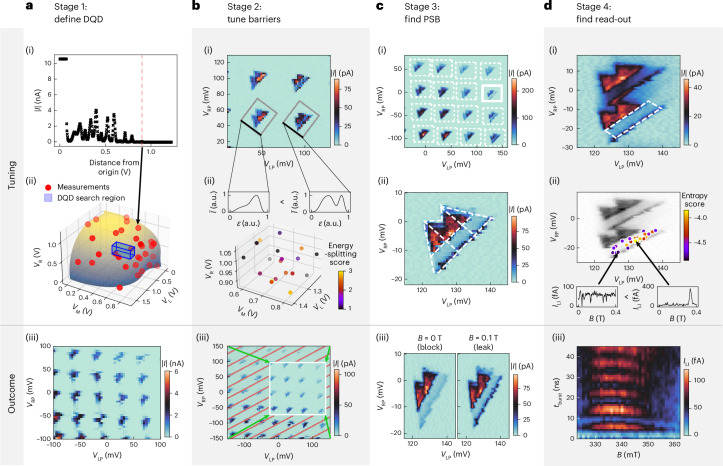
Summary of each stage. **a**, Stage 1: definition of a DQD potential. (i) Current measurements along random directions in the gate voltage space to determine the points at which conducting and non-conducting regions meet—the so-called pinch-off points. (ii) GP model of the hypersurface after collecting sufficient pinch-off points. (iii) A neural network confirms the presence of a DQD analysing the acquired stability diagrams. **b**, Stage 2: optimization of the bias triangle features. (i) Analysis of stability diagrams, segregating individual transitions and averaging them along segments orthogonal to the detuning axes. (ii) Distribution of optimization scores and averages of bias triangles along segments orthogonal to their detuning axes. We aim to increase the singlet–triplet energy splitting via a proxy score that measures the dip in current between the baselines and the rest of the triangles. (iii) Identification of plunger voltage windows unaffected by charge switches aided by neural networks. **c**, Stage 3: finding PSB. (i) Initial low-resolution, wide-range detection of PSB using neural networks. (ii) Detailed high-resolution scans using segmentation algorithms. (iii) Bias triangles that show signatures of PSB and can be used to optimize the read-out. **d**, Stage 4: read-out spot identification within a promising transition. (i) Acquisition of stability diagrams pulsing gate RP to locate the read-out region (indicated by the white dashed box). (ii) Entropy-based scoring of magnetic field traces within the read-out region, optimized through Bayesian methods. (iii) Rabi oscillations for different magnetic fields around the resonance condition to confirm the qubit operation. The measurements marked with *I*_LI_ were amplified with a lock-in amplifier (Methods).

Building on the methodologies of refs. ^21,22^, a hypersurface model is created to distinguish between conducting and non-conducting regions within the three-dimensional barrier gate voltage space. The algorithm takes current measurements along random directions within this space (Fig. 2a(i)), and models the hypersurface with a Gaussian process (GP; Fig. 2a(ii)). We expect a DQD potential forming near a corner of the hypersurface in the first octant. To pinpoint this corner, three specific current measurements are conducted using only one of the gate electrodes at a time. The resulting coordinates are then projected onto the model of the hypersurface, setting the lower bounds of the region in which DQDs are likely to be found. The upper bounds of the region are given by the coordinates of the single gate pinch-off voltages, that is, when the current drops to a value that is indistinguishable from the noise floor. The resulting box is labelled as ‘DQD search region’ in Fig. 2a(ii).

A methodical search within this box is conducted by the algorithm. The algorithm samples locations in the DQD search region and investigates them, starting from the point nearest to the projected corner and progressing to higher gate voltages. At each location, a one-dimensional trace of the plunger gate voltages is taken and checked for Coulomb peaks, a signature for quantized charge transport and the first requirement for a DQD. If Coulomb peaks are found, a measurement varying both plunger gate voltages (a so-called stability diagram) is acquired and analysed via a neural network for DQD characteristics (Fig. 2a(iii) shows a stability diagram with the desired features). Successful identification of DQD features at a location in the gate voltage space establishes it as a lower limit for the subsequent search.

The goal in the second stage is to adjust the tunnel barriers to enhance the singlet–triplet energy splitting, an important requirement for qubit operation. Additionally, this stage needs to avoid regions of gate voltage space with the following characteristics: regions with high currents above a generous threshold, regions that are susceptible to charge switch noise and regions that show co-tunnelling lines.

**Extended Data Fig. 2:**
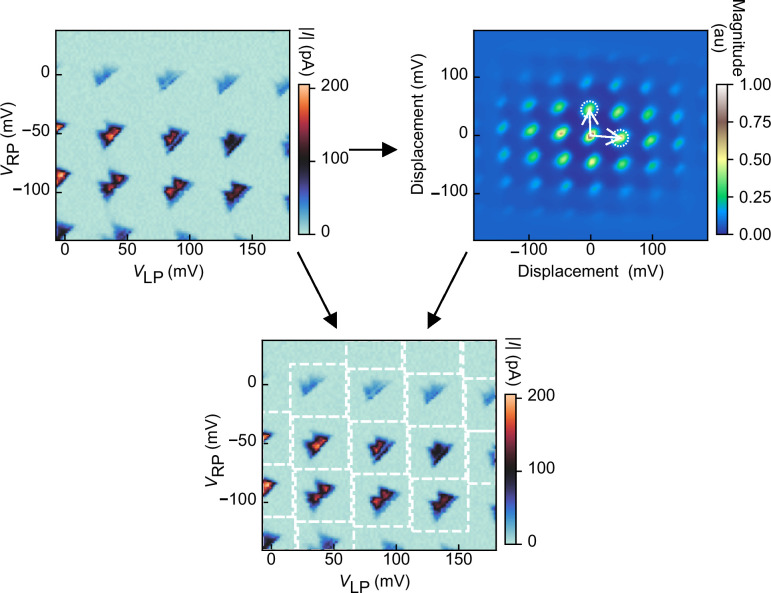
Bias triangle locations via auto-correlation. A stability diagram (top left) is processed using its auto-correlation (top right). Within the auto-correlation, we can find the highest values to span a grid of skewed rectangles. The extracted information is used to locate bias triangles in the original stability diagram (bottom).

Within the gate voltage bounds established in the first stage, Bayesian optimization is used to search for gate voltage combinations. The figure of merit for this optimization is based on the degree to which it reduces current between the triangle baseline and excited states, compared with the current throughout the remaining bias triangles. This acts as an easy-to-compute proxy score of the singlet–triplet energy splitting. The score is further detailed in the ‘Stage 2: tune barriers’ section. After Bayesian optimization suggests a voltage setting, a stability diagram is measured (Fig. 2b(i)). We first use a segmentation algorithm^49^ to find the outlines of individual pairs of bias triangles. The score is then computed using averages of the stability diagram along the common baseline (Fig. 2b(ii)). A neural network identifies bias triangles that are impacted by charge switches. Charge switches distort the stability diagrams and make the area unsuitable for qubit operation (Fig. 2b(iii) (hatched area) and Extended Data Fig. 2). These bias triangles are excluded from optimization. The gate voltage regions explored by optimization are shown in Fig. 2b(ii).

A substantial challenge of this stage is the need for numerous stability diagrams, which are time intensive to measure. To address this, we implement an adaptive, efficient measurement algorithm designed to specifically focus on gate voltage regions in which the bias triangles are present (Supplementary Section 6). Using this method cuts down the measurement time by approximately two-thirds. The optimization is performed using these efficient measurements.

As the final step, stability diagrams without the efficient measurement algorithm are taken in the most promising regions. This is done to ensure that there are no charge switches, because the previous step ranked each location by the highest score of a bias triangle at that location. Therefore, some stability diagrams may have regions affected by charge switches. Bounding boxes are then created in the plunger voltage space, encompassing primarily stable bias triangles with current below the previously mentioned threshold (Fig. 2b(iii)). These triangles are each evaluated and scored, and the bounding boxes are ranked based on the highest score they contain.

Up to this point, the algorithm has only used a positive bias voltage. This stage proposes both positive and negative bias voltages for each candidate it creates. The gate voltage coordinates including the bias voltage are passed onto the subsequent stage.

In the third stage, our algorithm searches for charge transitions exhibiting PSB, a necessary condition for qubit initialization and read-out in this setup. A candidate has to pass three different classifiers to be judged as exhibiting PSB. This is necessary to avoid false positives entering the time-intensive last stage.

We begin with low-resolution stability diagrams of the bias triangle candidates, both with *B* = 0 T and *B* = 0.1 T. In these devices, PSB is expressed as a suppressed baseline of the bias triangles in a low magnetic field compared with high magnetic fields (Fig. 2c(iii)). A comparison between the positive and negative bias voltages could also be used at the benefit of removing slow magnetic field sweeps but at the cost of less-reliable signatures.

**Extended Data Fig. 3:**
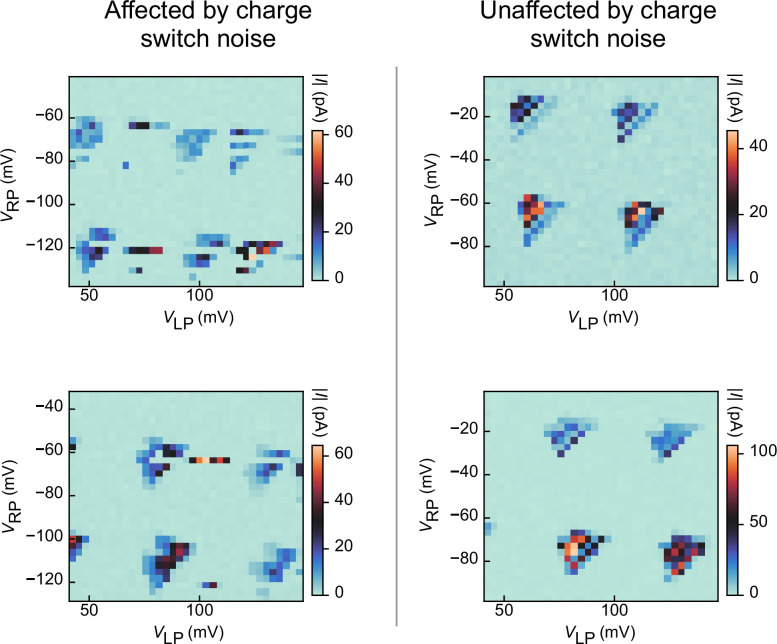
Effect of charge switch noise. Stability diagrams can be affected by charge switches. Their effect can be seen in the left column. For comparison, the right column is unaffected by these charge switches.

We use a routine based on the autocorrelation of the stability diagram to pinpoint bias triangle locations (Methods and Extended Data Fig. 3). The identified triangles are then analysed using a neural network^38^(Fig. 2c(i)). Subsequently, the algorithm measures a stability diagram to precisely delineate the bias triangle, followed by high-resolution stability diagrams with *B* = 0 T and *B* = 0.1 T.

**Extended Data Fig. 4:**
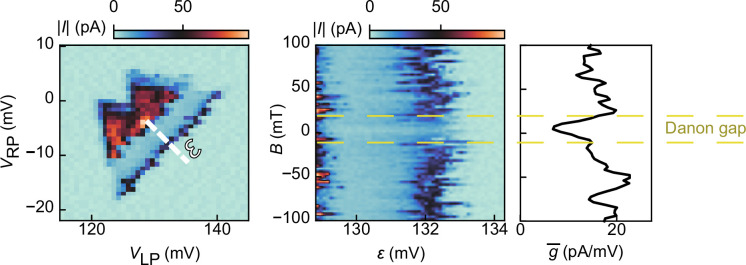
Danon gap check. As a final check to confirm the presence of PSB, the algorithm takes measurements of the detuning line *ε* (marked in the left panel) as a function of the magnetic field *B* (middle). The measurement is analysed by first computing the averaged sum of absolute derivatives ${\bar{g}}$ (right). If a dip is present in ${\bar{g}}$ close to *B* = 0 T, PSB is confirmed.

The algorithm invokes a routine to segment the bias triangles^49^ (Fig. 2c(ii)). A further PSB classification based on the segmentation is performed. The routine from ref. ^49^ defines the direction in the plunger voltage space that controls the detuning *ε* of the dot energies, known as the detuning axis of the bias triangles. Scanning along this line with varying magnetic fields, we expect to observe a current drop at the baseline at zero magnetic field, which another classifier detects (Extended Data Fig. 4). On meeting all criteria, the gate voltage coordinates are forwarded to the next stage.

The final stage of the process is dedicated to finding an operating point for qubit read-out and manipulation. This stage not only identifies a suitable location in the plunger voltage space but also determines the optimal driving frequency and duration of the pulse.

On the basis of the segmentation from the previous stage, a predicted read-out gate voltage region within the bias triangle is defined (Fig. 2d(i)). The algorithm optimizes across the four-dimensional space of the two plunger gate voltages, driving frequencies and pulse durations. It samples a point within this space and then measures the current as a function of the magnetic field (Fig. 2d(ii)). The optimization’s goal is to identify a current peak in these scans, indicative of the qubit’s resonance condition (Fig. 2d(ii), bottom-right plot). This is achieved by evaluating the entropy of current traces; traces exhibiting a peak correspond to lower entropy values (Methods).

Bayesian optimization then proposes potential candidates for further analysis. These candidates are filtered based on the presence of one or two peaks (corresponding to the number of qubits addressed), as determined by a simple peak-finding algorithm^50^. Noisy measurements might also show peaks. Therefore, a follow-up step involves retaking the measurement to confirm the presence of a peak.

**Extended Data Fig. 5:**
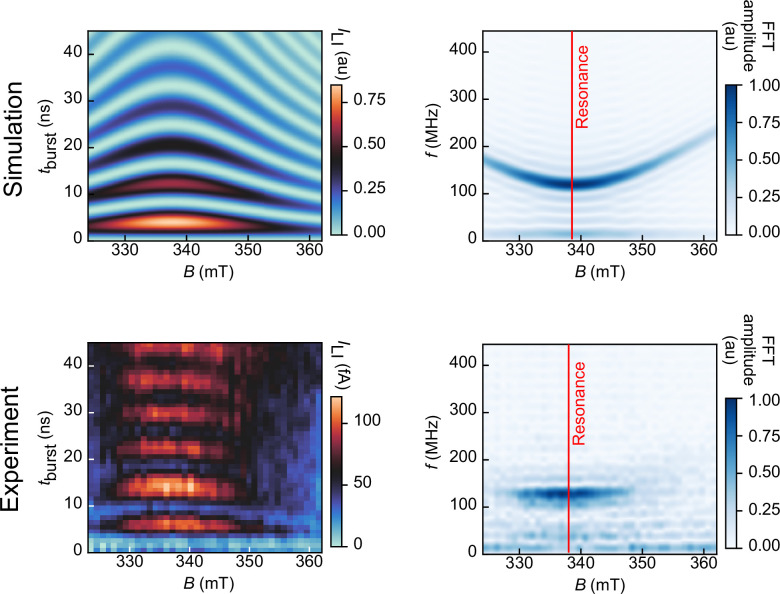
Rabi chevron analysis. A qubit that is driven close to the resonance condition exhibits so-called Rabi chevrons, see left column for examples (top: simulation, bottom: experimental data). To find the resonance frequency, we analyse the frequency spectrum for each magnetic field (which controls the detuning from resonance). The frequencies have a maximum amplitude and a minimal point at the resonance condition.

Once a candidate is verified, several measurements are taken by the algorithm to establish the qubit’s operational functionality. These include a spectroscopy measurement; a Rabi chevron experiment (Fig. 2d(iii)), which is used to calibrate the resonance frequency (Extended Data Fig. 5); and repeated high-resolution Rabi oscillations at the Larmor frequency.

Each stage requires a set of hyperparameters. They control various aspects of the measurements such as resolution and safe gate voltage ranges for stability diagrams; aspects of the signal processing algorithms such as the required prominence of peaks; and steering parameters such as the number of candidates that a stage can suggest. The measurement aspects can be derived from some weak prior knowledge about the device, such as the magnitude of lever arms, which informs the resolution of stability diagrams. We also assume a *g*-factor larger than 0.5 (limiting *B* and *f*_MW_) and *f*_Rabi_ between 30 MHz and 250 MHz (limiting *t*_burst_). The requirements on prior knowledge can be easily softened by widening the search space.

The set of hyperparameters influences the length of the runs and the way the algorithm manages trade-offs between exploration and exploitation. Regardless of the hyperparameters, the algorithm will always terminate once all the candidates of each stage have been exhausted.

The choice of hyperparameter was made during development and not optimized for the total run time or efficiency. We provide a full list of all hyperparameters in Supplementary Section 5.

After a qubit has been found, we may choose to study the qubit further. Routines from stages 3 and 4 enable the tracking of a known read-out spot and the resonance condition in the gate voltage space. This allows for recording the dependency of qubit metrics as we change the confinement potential. We provide details of this characterization algorithm in the Methods.

## Tuning performance

**Fig. 3:**
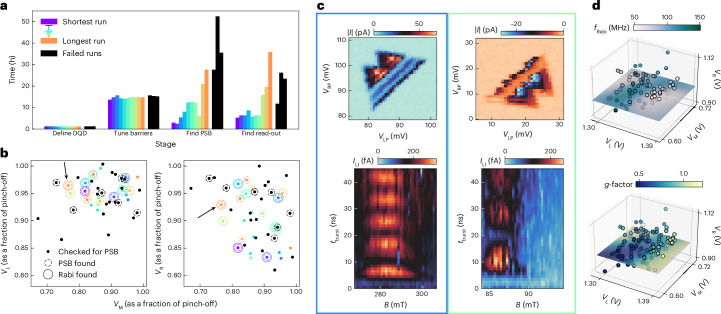
Benchmarking. **a**, Duration of each stage for ten successful runs is shown, sorted by the total time taken to tune a qubit in operation. Black: the three runs that did not lead to a qubit. **b**, Locations in the barrier voltage space (as a fraction of the individual pinch-off voltage) is shown. In some locations, no PSB was identified. The qubit locations are a subset of locations that exhibit PSB. In the failed runs, PSB was found but no Rabi oscillations were observed. **c**, Examples of transitions from two different runs, showing the associated bias triangles (top row) and Rabi chevrons (bottom row). **d**, Characterization. By varying the barrier gate voltages, we can build a map of *f*_Rabi_ (top row) and *g*-factor (bottom row) of one of the found qubits (marked with arrows in **b**) using automated measurements. For illustrative purposes, we show slices of a GP model that were fitted to the data.

We fixed the hyperparameters and gathered 13 runs. Rabi oscillations were found in ten of those runs. In successful runs, the time spent in each stage varied (Fig. 3a). The total time required ranged from 22 to 80 h, with a mean of 38 h (median, 34 h; Table 1). Each stage relies on the exploration and accuracy of the previous stage. The variation in the time required in each stage gets progressively larger. Almost all time is spent on measuring the device, not on the decision algorithms. This is due to the measurement of current through the DQD, which requires long integration times. A setup that allows for fast measurements via, for example, radio-frequency reflectometry could be tuned orders of magnitudes faster.

**Table 1:** Metrics of successful runs

Run number	Time	*f*_Rabi_	*g*	*V*_L_	*V*_M_	*V*_R_
	(h)	(MHz)		(V)	(V)	(V)
1	22.8	31 ± 1	1.52 ± 0.07	1.34	0.69	0.91
2	24.5	97 ± 2	0.72 ± 0.05	1.34	0.81	1.02
3	28.5	109 ± 1	0.70 ± 0.05	1.34	0.79	0.95
4	32.0	47 ± 1	2.20 ± 0.12	1.37	0.80	1.01
5	33.3	51 ± 1	2.74 ± 0.22	1.36	0.69	1.04
6	34.4	90 ± 1	0.73 ± 0.04	1.35	0.78	0.94
7	34.5	56 ± 1	2.31 ± 0.14	1.35	0.79	0.95
8	37.8	115 ± 3	0.67 ± 0.08	1.32	0.77	0.89
9	56.5	49 ± 1	2.12 ± 0.14	1.35	0.65	1.00
10	79.2	63 ± 1	0.74 ± 0.03	1.38	0.71	1.02

For each qubit found, we show the total time it took; Rabi frequency *f*_Rabi_; *g*-factor; and settings for barrier gates *V*_L_, *V*_M_ and *V*_R_. The errors of *f*_Rabi_ are estimated from the fit uncertainty and the errors of *g* are calculated from the width of the resonance peak.

The three failed runs ultimately reached a limit on the explorations it is allowed to make, terminating the search to prevent an unbounded exploration of parameter space. They lasted between 56.0 h and 94.9 h (Fig. 3a, black). Although these runs identified transitions consistent with PSB, the algorithm could not proceed to detect Rabi oscillations. Since the optimal conditions for qubit formation are not uniformly distributed, the algorithm can sometimes converge on parameter regimes in which the tunnel-barrier settings are less optimal. Additionally, when the signal-to-noise ratio is lower, the algorithm may not yield a positive detection.

The qubit locations in the gate voltage space in terms of the three barrier gate voltages are depicted in Fig. 3b. For comparability, we normalize each voltage by the voltage at which each barrier gate electrode pinches off the current individually. At each point, stage 2 (tune barriers) passed a candidate for further analysis (solid dots). In some cases, PSB was detected (dashed circles), passing stage 3, and a subset of these also yielded a qubit (solid circles), successfully completing stage 4. A similar plot showing the distribution in plunger voltage space is provided in Supplementary Section 2 and Supplementary Fig. 2.

Figure 3c presents examples from two runs, showing the transitions found and Rabi chevron measurements. The discovery of qubits in both bias directions evidences the algorithm’s adaptability and its non-specificity to certain transitions. Both Rabi chevrons were obtained using the same given driving power and driving frequency, but varied in magnetic fields and Rabi frequencies, highlighting the algorithm’s generalization capability. All the depicted measurements were autonomously executed by the algorithm, accounting for the non-centred chevron measurements. Respective measurements for all successful runs are provided in Supplementary Fig. 1.

Analysing the locations in the gate voltage space in which qubits were found provides insight into the device physics (Table 1). By fitting a convex hull around the qubit locations in the barrier gate voltage space, we can estimate the volume of the region in which qubits can be found. For this device, the volume of the convex hull is approximately 3.2 × 10^−4^ V^3^, translating to a fraction of the safe ranges ((2 V)^3^) of about 4 × 10^−5^. The space is further restricted by the plunger voltage location, which is a box of roughly (10 mV)^2^. Given a search space of (300 mV)^2^, this brings down the size of the volume to around 2 × 10^−7^ as a fraction of the five-dimensional gate voltage space, which is roughly equivalent to a needle in a (2 m)^3^ haystack.

Once a qubit has been found, the algorithm allows for extensive characterization. We can study *f*_Rabi_ and *g*-factor as a function of the barrier gate voltages (Fig. 3d). The resulting maps give insights into qubit properties and can be extended to measure, for example, the Hahn-echo coherence time.

## Conclusions

We have reported the fully automated tuning of spin qubits, progressing from a de-energized device to qubit control. Our algorithm autonomously achieved Rabi oscillations in 10 out of 13 trials. Most tuning processes concluded within three days, with the primary speed constraint being the integration times required to perform d.c. transport measurements. The times can be sped up via fast read-out alternative or spin manipulation techniques of the quantum device. Maps of the *g*-factor and *f*_Rabi_ serve as evidence for the potential of this approach for high-throughput qubit characterization.

Our methodology could be extended to other semiconductor-based qubit architectures, including silicon fin field-effect transistors^15^. The modular design of our algorithm makes adapting to different device layouts and measurement techniques—including charge sensing and single-shot read-out—accessible. Although certain modules and optimization approaches may require refinement to suit larger or more complex devices, our end-to-end approach provides a foundation for future work.

Our autonomous pipeline could also be extended beyond qubit formation towards high-fidelity control. Additional stages can be added that automatically design and refine shaped control pulses, an approach that has already pushed single- and two-qubit fidelities past fault tolerance thresholds when performed manually^51–53^. Coupling such pulse engineering routines with the gate voltage tuning demonstrated here, as well as scaling the full loop to multiqubit architectures, defines the next step for fully self-optimizing spin qubit hardware. Meeting these challenges will be pivotal for realizing hands-free, high-performance quantum processors. More broadly, we expect the internal scores that steer our autonomous loop, such as energy-splitting and entropy measures, may correlate with established qubit-quality benchmarks; systematically mapping those connections, as well as refining the scores accordingly, is a promising direction for future optimization studies.

The mass tuning and characterization of qubits, facilitated by our fully autonomous tuning algorithm, could provide a productive feedback loop between measurement and fabrication processes. Wafer-scale, high-throughput characterization of quantum devices, already feasible in early tuning stages, can mitigate device variability. This, in turn, could improve the tuning process, bolstered by expanding datasets.

## Methods

Here we provide details of the experimental setup and the algorithmic choices made in this study. We begin by describing the device used for our experiments. We then follow with a discussion of the intelligent computational approaches used in the algorithm. Finally, details of each stage are discussed. An overview of all the stages of the algorithm is given in Extended Data Table 1.

Hyperparameters that are not explicitly given here are provided in Supplementary Section 5.

### Device and measurement details

The device consists of a Ge–Si core–shell nanowire lying on top of nine bottom gates measured in a variable-temperature insert in a liquid-helium bath, with the sample mounted below the 1-K pot (base temperature, 1.5 K). By applying positive voltages to the first five bottom gates from the left, an intrinsic hole gas inside the nanowire is depleted to form a hole DQD. A scanning electron microscopy image of a device similar to the one used here is shown in Extended Data Fig. 1. The device, measurement apparatus and pulse sequence are the same as those used in ref. ^41^ and are described in detail in that work, which was performed independently and served primarily as an initial reference for the device’s characteristics. We did not explicitly encode knowledge of the particular qubit from ref. ^41^ into our algorithm.

To amplify the measurements that rely on an MW pulse, we applied pulse modulation by a lock-in amplifier at 87.777 Hz. The measurements, therefore, have an in-phase and out-of-phase component. We apply principal component analysis to these measurements and project each measurement onto the principal axis. We further offset the measurements such that they are strictly positive. Measurements that are obtained in this way are marked as *I*_LI_, as opposed to currents that were measured conventionally (which are marked as ∣*I*∣).

### Techniques used in the algorithm

Useful automation of tuning from a de-energized device to identify Rabi oscillations requires an algorithm that can adapt to different data-capture regimes, and be transferred to other, similar devices. To achieve this, we have made extensive use of intelligent, adaptive and data-driven subroutines. Nowadays, there is a plethora of such techniques to choose from. To choose the right technique to apply to each stage of the algorithm, we considered the following:

- the need for expert-labelled training data, which was not always possible or realistic to source;
- the need of being efficient in both total number of measurements taken during a stage and in the resources needed for computing decisions about which measurements to take;
- the minimal accuracy needed in each stage for the whole algorithm to be able to achieve its overall goal of identifying operating parameters for a qubit.

To address the above considerations across the many stages of the algorithm, a non-exhaustive list of techniques we have found useful to use includes GP inference, convolutional neural networks (CNNs), unsupervised computer vision and computational geometry techniques, and Bayesian optimization. We now briefly introduce these, highlighting their strengths and weaknesses.

GPs are a popular form of non-parametric Bayesian inference^54^. They can be thought of as a method for doing principled Bayesian inference over a space of functions. GPs can be tailored to any specific domain or problem by making a choice of the so-called kernel (or covariance) function, a part of this model that describes prior knowledge about the possible space of functions in which inference is to occur. This choice can allow practitioners to encode important domain knowledge before capturing any data, such as specifying knowledge of periodicities, symmetries or the expected degree of smoothness of the underlying process that is being observed. This constitutes the main strength of GP modelling, often enabling highly data-efficient inference. However, both model fitting and model prediction can be computationally intensive, typically growing cubically^54^ in the number of observed data points.

Over the past two decades, CNN architectures have proved to be go-to models for solving computer vision tasks. Their strengths lie in their adaptability across different computer vision tasks, their robustness in the face of unknown noise and their computational efficiency at training time. Their weakness lies in always requiring substantial amounts of training data. In this work, we use some standard architectures, such as ResNet^55^, as tools for extracting properties from or making assessments of stability diagrams. Where we have applied them, training data have been either generated by a sufficiently good simulator or gathered from this or similar devices and then labelled by an expert.

Often, the use of CNNs is neither required nor appropriate for the particular computer vision task at hand. In particular, it has been of crucial importance to a number of stages through the algorithm to be able to automatically locate and segment bias triangles within stability diagrams using a coordinate-wise approach. To achieve this, we have used a number of unsupervised computer vision techniques that can mitigate noise in, and localize features of the geometric figures present in the stability diagrams. Although CNNs would require large amounts of labelled training data to achieve this result, the requirements can be met by computer vision techniques that need no training data, and require only a few hyperparameter choices to be made. All together, we have called this a bias triangle segmentation framework, and it is described in ref. ^49^, where the specific application to PSB detection is also detailed.

Bayesian optimization^21,22,56,57^ is a general, iterative approach to black-box function optimization. At each iteration, it constructs a surrogate model of the function being approximated using the data already gathered, and uses this surrogate model to efficiently compute the next most informative location from which to sample the unknown function. To apply this technique, one must specify a score function to optimize and a parameter space over which the search for an optimum is conducted. The choice of the surrogate model is also influential in the accuracy, efficiency and reliability achieved using this method. In this work, we have made consistent use of GP surrogate models using a Matern 5/2 kernel.

### Stages of the algorithm

#### Stage 1: define DQD

(a) Hypersurface building. As the first step, the algorithm determines a current that it considers to be pinched off by ramping to the high end of the safe ranges. We take repeated current measurements there to characterize the noise floor. From the noise floor, we compute a pinch-off current.

Next, we sample several points within the safe ranges using a Sobol sequence for quasi-random locations. The points are used to define rays from the origin that are then investigated for pinch-off. To avoid overloading the current amplifier, we search for pinch-off from the origin towards the upper end of the safety ranges with a low bias voltage. Once the pinch-off is found, we retrace with a higher bias voltage to confirm the exact pinch-off location.

Finally, we use these data to construct the hypersurface model, as outlined in the main text.

(b) Double-dot detection. The previous stage defines a region within which we can look for a DQD potential. We sample quasi-random points via a Sobol sequence for investigation. For each point, we vary both plunger voltages simultaneously and measure the current, following the method described in ref. ^21^. We use the random forest classifier developed in ref. ^22^ to check for the presence of Coulomb peaks. If Coulomb peaks are found, we then measure a stability diagram. This diagram is analysed with a neural network to detect features of the DQD. The neural network was trained on data from a variety of devices, mainly from the data gathered in refs. ^21,22^, and additional data are obtained from a nanowire device different from the one used in this work. In total, there were 4,611 stability diagrams, out of which 726 showed double-dot features.

#### Stage 2: tune barriers

(a) Barrier optimization. On the formation of separated pairs of bias triangles, we perform coordinate-wise segmentation and polygon fitting^49^ to facilitate their tracking and feature extraction throughout the remainder of the tuning pipeline.

Segmentation and shape extraction enable the assessment of the current intensity difference between the baseline and gap formed between the base and first excited-state line. By quantifying this intensity difference in a base separation score, we can use a Bayesian optimization framework, which seeks to maximize the intensity difference, as a promising indicator for detecting viable candidates for PSB.

Each pair of bias triangles should present a base well separated from the main body. By measuring the d.c. current through the device, we effectively probe the internal energy transitions between the two quantum dots. Crucially, these transitions remain relatively unaffected by the thermal broadening of the leads, making the scheme viable at higher (‘hot’) temperatures. For PSB-based read-out to be effective, the energy splitting between the relevant singlet and triplet states, that is, *Δ*_ST_, must exceed the thermal energy (*k*_B_*T*). Once *Δ*_ST_ ≤ *k*_B_*T*, the PSB mechanism can degrade and allow unwanted leakage current, thereby compromising the quality of the read-out. The energy-splitting score, therefore, serves as a metric to guide the algorithm towards bias triangles that are more likely to yield robust spin read-out and, ultimately, stable qubit operation. Supplementary Section 3 provides further discussion on the relation of this score with spin-state visibility. Supplementary Section 4 provides empirical justification of the score.

The separation score is computed by averaging the current along the detuning axis (Fig. 2b(ii) shows two one-dimensional traces), and computing the ratio of the intensity between the peaks and the lowest point in the valley between them. In the case of multiple triangles, the highest separation is used as the score.

Certain potential landscapes can lead to situations in which charge configurations are affected by charge switch noise. Since these potentials are unlikely to be used as a qubit and the resulting bias triangles can skew our base separation scoring, we excluded them by leveraging a neural network classifier. This classifier was trained to distinguish between normal bias triangles and the ones that are affected by charge switch noise (Extended Data Fig. 2). The training dataset for this classifier was obtained as follows: initially, potential bias triangles were identified using our segmentation routine. Subsequently, we manually labelled 2,302 of these (1,539 samples showed no switch noise) to create a robust training set. The classifier itself was then obtained by fine-tuning a ResNet-based architecture with this dataset.

Once the voltage space has been explored through Bayesian optimization, we have a clear understanding of the landscape (Fig. 2b(ii)). The measurements in this optimization were obtained using an efficient measurement algorithm (Supplementary Section 6). We sample the most promising regions again without the efficient measurement algorithm and analyse them, as described in the next section.

(b) Plunger window selection. Given a sampled stability diagram containing bias triangles, the aim is to select the region that contains as many bias triangles with scores as high as possible, no areas with current that is too high and as few switches as possible (Fig. 2b(iii)). High-current bias triangles are unlikely to be able to be used for qubit operations. This is a heuristic and we set a conservative threshold of 200 pA. To ease the downstream steps, the region should be a rectangular window. Through an iterative approach, starting from the smallest bounding boxes containing each single pair of bias triangles, larger windows are constructed by merging the existing ones in case they satisfy the conditions about switch absence and low currents. For the absence of switches, we used a soft constraint, allowing for bias triangles affected by switch noise in case their total area was less than 25% of the area covered by all triangles in the window. The algorithm complexity scales exponentially with respect to the number of triangles and some heuristics have been leveraged to reduce substantially complexity and, therefore, execution time. In particular, at each iteration, only the top 100 bounding boxes by the number of contained triangles without switches were kept, to ensure a manageable upper limit on the number of possible merges. The routine halts when no further merges are possible. Once the plunger windows have been selected, they are ranked by the highest separation score.

#### Stage 3: find PSB

(a) Wide-shot PSB detection. To identify each bias triangle’s location, we first leverage the fact that they sit on a honeycomb or skewed rectangle pattern. We use autocorrelation on the stability diagram to identify this pattern. The largest two peaks in the autocorrelation help us establish a vector that spans this pattern of skewed rectangles. To fix the pattern in place, we use a blob detection algorithm, using the first blob it identifies. This helps us accurately overlay the skewed rectangle pattern and estimate the locations of the bias triangles (Extended Data Fig. 3).

Next, we extract these bias triangles using the identified locations, with side lengths informed by the pattern dimensions. These extracted bias triangles are then input into a neural network for further analysis. We used autonomously gathered data that were taken during the initial development phase. In total, we used 626 pairs of bias triangles taken from 70 stability diagrams. Here 55 pairs of bias triangles showed PSB. Reference ^38^ provides more detailed information on this procedure.

(b) Re-centring and high-resolution PSB detection. In an effort to filter the previously detected candidates for PSB and eliminate false positives, a second set of higher-resolution measurements is performed. For that purpose, a dedicated low-resolution stability diagram of the candidate bias triangles is taken and used to update the plunger voltage extent based on the detected contours, effectively performing a re-centring. With the updated voltage extent, high-resolution stability diagrams with *B* = 0 T and with *B* = 0.1 T are taken.

A second substage of PSB classification is applied through a segmentation-based detection and feature extraction framework, which facilitates the coordinate-wise quantification of geometric and physical properties of bias triangles^49^. In particular, given the high-resolution stability diagram with *B* = 0.1 T, this framework fits minimum-edge polygons to the detected contours of bias triangle pairs by utilizing a relaxed extension of the Ramer–Douglas–Peucker algorithm^49^. Once the segmented shape mask is identified, further geometric properties such as the base and excited-state lines can be automatically extracted solely based on prior knowledge of the bias voltage sign, which predicts the direction in which the bias triangles point.

For the identification of PSB, an analytical classifier based on the above framework was devised^49^. PSB expresses itself as a suppressed baseline of the bias triangles at *B* = 0 T. At *B* > 0 T, there is a leakage current at the baseline. The routine extracts the segment enclosing the base and a prominent excited-state line on the stability diagram with a leakage current (*B* = 0.1 T). Subsequently, the average pixel intensity of the segment normalized by the intensity of the entire pair of bias triangles is computed. By superimposing the detected segment on the scan with blocked current (*B* = 0 T), normalized intensity values are compared and, if their difference exceeds a specified threshold, the charge transition is identified as positive for PSB.

On the basis of the segmented shape mask of the bias triangle, further geometrical properties can be automatically extracted, which enables the tuning of bias triangle features in stage 2. The detuning axis, utilized for the Danon gap measurement, is automatically extracted by identifying the bias triangles’ base midpoints and tips and computing the lines between them.

(c) Danon gap check. As a further filter for possible candidates, we check to find the magnetic field dependence of the leakage current at the base of the bias triangles in a different way. As a function of the applied magnetic field *B*, we expect the leakage current to be minimal at *B* = 0 T and higher away from this point. We call this the Danon gap^58^. A current measurement of the detuning line as the magnetic field is varied, giving us a two-dimensional input (Extended Data Fig. 4), which we can analyse as follows. Ignoring the noise signal, the current is roughly constant along the magnetic field axis, whereas the detuning line axis is information rich. Away from the Danon gap, there are two local extrema (to one side, the noise floor outside the bias triangles, and to the other side, the gap due to singlet–triplet energy splitting), whereas the Danon gap region is characterized by a monotonic behaviour, with roughly a constant value.

To detect the presence of the Danon gap, the current *I* is first processed with a Gaussian filter, to smooth out the noise, and then the absolute slopes along the detuning line axis are integrated: $g(B):={\sum }_{\varepsilon }\left|\frac{\partial \widetilde{I}(\varepsilon ,B)}{\partial \varepsilon }\right|$, where the derivative is the discrete derivative along the detuning line axis. The function *g* is minimized in the areas where the smoothed signal $\widetilde{I}$ shows a constant value. We show the normalized function ${\bar{g}} =\frac{g}{| \varepsilon | }$ in Extended Data Fig. 4. To detect the presence of the Danon gap from *g*, two tunable hyperparameters are used, validating the depth and width of the basin of the global minimum of *g*: in case the basin is not prominent enough, there is no Danon gap. As the last check, the location of the minima has to be in proximity of zero magnetic field.

#### Stage 4: find read-out

(a) Tracking and entropy optimization. In the subsequent steps, we apply a pulse sequence to the right plunger electrode. As it is a two-stage pulse, the bias triangles will have a ‘shadow’ in the stability diagram. We need to identify the original bias triangles and find a suitable region in which we can expect to find qubit read-out. In light of the resulting shape distortions and further degrading effects to the measurement quality, we opt for template matching as opposed to performing re-segmentation for bias triangle tracking to ensure robustness.

The relative direction in which the shadow bias triangles appear with respect to the original one is known in advance due to the applied pulse shape. This is incorporated into the shape-matching approach as the cardinal direction to uniquely identify and track the triangles. We perform shape matching by comparing the edge map of a stability diagram before pulsing, functioning as the template, to the edge map of a subsequent stability diagram with pulsing, functioning as the source for current information. Further, we extract the segmented shape mask from the template. The method slides the template over the source edge map, thereby comparing the template with individual patches of the stability diagram with pulsing, and returns a result matrix (of the same size as the source) whose individual entries quantify the similarity with the template patch. The used similarity metric is the normalized correlation coefficient, and the patch with the maximum correlation is selected. Once the appropriate patch has been identified, the initial segmentation mask of the stability diagram without pulsing is mapped to the stability diagram with pulsing and used for subsequent processing.

To identify the optimal read-out spot, we extract the segment enclosing the base and prominent excited-state lines on the obtained segmented mask of bias triangles with pulsing. We then perform Bayesian optimization of a read-out quality score over the following parameters: the constrained two-dimensional plunger gate voltage space, frequency of the driving pulse *f*_MW_ and burst time *t*_burst_.

Optimal read-out candidates are those that meet the resonance conditions of the qubit. If they are met, there is a leakage current that we record using the lock-in amplifier. For a given burst time *t*_burst_ (relating to the Rabi frequency *f*_Rabi_) and a given frequency of the driving pulse *f*_MW_ (relating to the *g*-factor), the leakage is characterized as a peak in leakage current for a certain magnetic field *B*. In this setup, hardware-related resonances and non-uniform attenuation across certain frequency ranges introduce distortions when the drive frequency is varied instead of the magnetic field. Consequently, sweeping the magnetic field at a fixed frequency provides a more stable and interpretable signal, even though it is slower. Thus, for read-out optimization, we measure the current with varying magnetic fields. Instead of applying principal component analysis, as explained above, we use the L2 norm of the in-phase and out-of-phase components to retrieve a one-dimensional trace *l*(*B*). This guarantees peaks to be higher than the background, as opposed to processing with principal component analysis, which can lead to dips rather than peaks.

To quantify the sharpness of these peaks, we developed a score based on the Shannon entropy $H=-{\sum }_{B}\left[l(B)\log [l(B)]\right]$ of the trace. For the calculation of the entropy of the score, we first subtract the median and then clip values at zero. This particular preprocessing turns the trace into something more akin to a distribution and enhances the robustness of our score, making it less susceptible to potential noise disturbances in the trace. This method results in a smooth score landscape suitable for Bayesian optimization.

(b) Resonance confirmation. This verification step acts as a final filter and all the last stages are executed once a candidate passes this filter. The previous stage sends a candidate with a suspected resonance condition. The stage re-measures the leakage current as a function of the magnetic field. If the resonance condition was truly found, a peak should appear at the same magnetic field again. If we detect a peak with a specified prominence at this magnetic field within a set margin of error, the resonance condition is considered confirmed and all downstream measurements are executed. We note that a noisy candidate might pass this stage. Repeating the verification step can reduce such occurrences.

(c) Qubit measurements. Once a resonance condition is found, we vary the magnetic field and burst duration. The characteristic Rabi chevron can be analysed by considering the frequency spectrum for each magnetic field. The frequency should have a minimum at the magnetic field that meets the resonance condition of the qubit. The amplitude should also be the maximum there due to decoherence for off-resonant driving. We can, therefore, simply look for the maximum amplitude in the Fourier-transformed Rabi chevron (Extended Data Fig. 5). This information will give us the precise resonance conditions for the last step, which are repeated measurements of Rabi oscillations on resonance.

### Characterization

The maps shown in Fig. 3 were generated using automated measurements. Initially, on identifying a qubit, we record its read-out spot, *g*-factor and *f*_Rabi_. We then alter the confinement potential by slightly adjusting the barrier gate voltages. This adjustment may shift the bias triangles, consequently moving the read-out spot, *g*-factor and *f*_Rabi_. Our method involves tracing these transitions to locate the read-out spot in its new position. At this new location, we conduct an electric dipole spin resonance check scan. Any changes in the peak’s location inform us about variations in the *g*-factor. Furthermore, measuring Rabi oscillations at this point helps update our understanding of *f*_Rabi_.

As we progressively deviate from the initial measurement point, we utilize our closest prior qubit data to infer the properties at the new location. This step is crucial as a shift in the *g*-factor necessitates modifying the magnetic field range, whereas a change in *f*_Rabi_ requires adjusting the *t*_burst_ duration for the electric dipole spin resonance check to accurately detect resonances.

## Supplementary information

Supplementary Information Supplementary Sections 1–8 and Figs. 1–7.

## Data Availability

The data are available via Zenodo (10.5281/zenodo.17745219)^59^.

## Extended data

**Extended Data Table 1:** List of all stages

Stage	Description	Techniques used	Candidate information
**Stage 1:** Define DQD			
**a**, Hypersurface building	Building a model of the surface that separates pinch-off from conducting	Gaussian processes	Upper and lower bounds of barrier voltages
**b**, Double dot detection	Identifying Coulomb peaks and double dot signatures	Random forests and neural networks	Corrected lower bound of barrier voltages
**Stage 2:** Tune Barriers			
**a**, Barrier optimisation	Search over the barrier voltage space for ideal settings	Bayesian optimisation, computer vision, neural networks	Promising barrier voltage location
**b**, Plunger window selection	Determine a window for plunger voltages	Computer vision, neural networks	Barrier voltage location and wide plunger voltage range
**Stage 3:** Find PSB			
**a**, Wide shot PSB detection	Identify locations of transitions with PSB	Neural networks, computer vision	Narrow plunger voltage range
**b**, Re-centering	Get precise plunger voltage window	Computer vision	Corrected narrow plunger voltage range
**c**, High res. PSB detection	Confirm PSB with high resolution measurement	Computer vision	Narrow plunger voltage range, link to high res. measurement, detuning line definition
**d**, Danon gap check	Confirm PSB by measuring detuning line as a function of magnetic field	Computer vision	Narrow plunger voltage range, link to high res. measurement
**Stage 4:** Find Readout			
**a**, Entropy optimisation	Find readout spot, *g*-factor and Rabi frequency by optimisation of an entropy score	Bayesian optimisation, computer vision	Precise plunger voltage locations, magnetic field, drive frequency and burst time
**b**, Resonance confirmation	Confirm resonance from previous stage/filter out noise	Peak finding	Passed on from previous
**c**, Spectroscopy	Measurement of current while varying *t*_burst_ and *f*_MW_ for documentation	-	Passed on from previous
**d**, Rabi chevron	Measuring Rabi oscillations close to the resonance condition	Frequency analysis	Corrected magnetic field for resonance condition
**e**, Rabi oscillations	Take repeated Rabi oscillations on resonance	-	-

We list all stages and sub-stages with a short description, a rough overview of what techniques were used and what information is passed downstream for a candidate from each stage. Besides information on the parameters that are directly needed to operate a qubit, the stages also pass down meta-information that other stages might need to use, for example the high resolution stability diagram of Stage 3c is needed in Stage 4a for template matching.

## Code availability

The code is available via GitHub at https://github.com/oxquantum-repo/fully-autonomous-tuning.
