# Supplementary material for: Fully autonomous tuning of a spin qubit
Source: Nat Electron. 2026 Feb 2;9(3):304–13. doi: 10.1038/s41928-025-01562-4 (PMC13035472; doi:10.1038/s41928-025-01562-4)
Supplement: Supplementary file 1 — Supplementary Sections 1–8 and Figs. 1–7. [file 41928_2025_1562_MOESM1_ESM.pdf]

nature electronics

Supplementary information

https://doi.org/10.1038/s41928-025-01562-4

Fully autonomous tuning of a spin qubit

In the format provided by the authors and unedited

CONTENTS

S1. Qubit measurements of all successful runs 2

S2. Distribution of runs in plunger-plunger space 2

S3. Relation of visibility to the energy-splitting and entropy scores 4

S4. Empirical justification for the energy-splitting score 5

S5. Hyperparameters 8
1. Define DQD 8
a. Hypersurface building 8
b. Double dot detection 8
2. Tune barriers 9
a. Entropy optimisation 9
b. Plunger window detection 9
3. Find PSB 9
a. Wide shot PSB detection 9
b. Re-centering 10
c. High resolution PSB detection 10
d. Danon gap check 10
4. Find readout 11
a. Entropy optimisation 11
b. Resonance confirmation 12
c. Spectroscopy 12
d. Rabi chevron 12
e. Rabi oscillations 13

S6. Efficient measurement algorithm 14

S7. Search tree examples 15

S8. Modular framework 16
1. Stage structure 16
a. Functions 16
2. Candidates 16

References 16

S1. QUBIT MEASUREMENTS OF ALL SUCCESSFUL RUNS

We show measurements that confirm that we found a qubit in the ten successful runs, ordered by total run time as in the main text, see Supplementary Fig. 1. The measurements were all taken autonomously. We show the associated pair of bias triangles (upper left in each panel), a measurement varying the magnetic field and the driving frequency (upper right), a Rabi chevron measurement where we vary the magnetic field and the burst duration (lower left), and an averaged measurement of Rabi oscillations (lower right) at the magnetic field indicated with dashed lines in the Rabi chevron measurement.

While the diversity in plunger voltage settings, magnetic field values, and Rabi frequencies underscores the versatility of our algorithm, we also observe that certain runs display similarities in gate voltages (e.g., [b,h], [c,f], [d,g] in Supplementary Fig. 1). One possible explanation is the even-odd filling pattern discussed by Johnson *et al.* [1] , in which the condition for observing Pauli spin blockade follows a "checkerboard" dependence on charge occupancy. In principle, applying virtual gates to correct for cross-coupling could group these qubits more systematically; however, subtle effects such as random charge rearrangements complicates such an analysis.

Furthermore, the data presented in Fig. 3d of the main text show how a single qubit can be studied using of our autonomous approach. The variation of observed g and f_{Rabi} demonstrates that our method systematically probes a broad array of operating parameters than those typically accessed through manual tuning. While human experts might also observe a similar spread in qubit frequencies, our automated approach can exhaustively scan the parameter space and identify subtle adjustments—such as slight modifications to barrier voltages—that lead to Pauli spin blockade (PSB) and viable qubit c onfigurations. These findings validate the performance of our algorithm and provide a valuable foundation for more detailed statistical analyses of qubit behavior in future studies.

S2. DISTRIBUTION OF RUNS IN PLUNGER-PLUNGER SPACE

Supplementary Fig. 2 complements Fig. 2b of the main text by showing where positively identified PSB candidates and fully tuned qubits were located in the two-dimensional plunger-gate plane.

a. Raw plunger coordinates. Panel **a** displays the uncompensated plunger voltages at which PSB (dashed circles) and qubits (solid circles) were observed. Each point is a projection of a five-dimensional gate vector, so the associated barrier-gate settings vary from run to run. For clarity, we omit the much larger set of plunger windows that were merely screened for PSB in Stage 3 but did not yield a positive classification. Symbols carry the same colour code as Fig. 2b (main text) and Supplementary Fig. 1 of this Supplementary Information; a "+" ("−") indicates that PSB or a qubit was found under positive (negative) bias.

Because barrier voltages differ between runs and cross-capacitive shifts are not accounted for, the raw distribution appears scattered.

b. Virtual plunger coordinates. To reduce this spread, we first measured the cross-capacitances between each barrier and plunger gate (e.g., a 100 mV change on the left barrier mimics a ∼ 50 mV shift on the left plunger). We then define virtual plunger coordinates that compensate this cross-talk and register all runs to a common plunger frame.

More precisely, we compute the virtualised plunger voltages (denoted $\tilde{V}_{\mathrm{LP}}$ and $\tilde{V}_{\mathrm{RP}}$) via

$$\begin{pmatrix}\tilde{V}_{\mathrm{LP}}\\ \tilde{V}_{\mathrm{RP}}\end{pmatrix} = \begin{pmatrix}V_{\mathrm{LP}}\\ V_{\mathrm{RP}}\end{pmatrix} - \mathbf{M}\begin{pmatrix}V_{\mathrm{L}} - V_{\mathrm{L,ref}}\\ V_{\mathrm{M}} - V_{\mathrm{M,ref}}\\ V_{\mathrm{R}} - V_{\mathrm{R,ref}}\end{pmatrix}, \qquad \mathbf{M} = \begin{pmatrix}0.521 & 0.521 & 0\\ 0 & 0.417 & 0.625\end{pmatrix}, \tag{1}$$

where V_{LP} and V_{RP} are the plunger voltages, $V_{\mathrm{L}}, V_{\mathrm{M}}, V_{\mathrm{R}}$ the barrier voltages, and $V_{\mathrm{L,ref}}, V_{\mathrm{M,ref}}, V_{\mathrm{R,ref}}$ the chosen reference barrier-voltage vector that sets the common display frame. The matrix **M** (calibrated experimentally) captures the linear cross-capacitances. We register each run by subtracting its barrier offsets relative to the reference vector.

We choose this virtualisation against a reference point/run to improve visual interpretability of the combined data. The linear model is an approximation: occasional charge rearrangements induce non-linear shifts that a fixed **M** cannot remove, so some residual mis-alignment is expected.

Panels **b** and **c** show the resulting compensated coordinates, each registered to the coordinate frame of a different successful run (third- and fifth-shortest, respectively). The background images in Panels **b** and **c** are the corresponding charge-stability diagrams of the reference runs, providing a sense of scale.

After compensation, several points collapse into tight clusters, demonstrating that multiple runs converged on the same charge transition. Nonetheless, compensation remains approximate: a persistent charge reconfiguration in the device induces non-linear shifts that cannot be removed by a simple linear virtual-gate matrix. Consequently, certain data pairs appear close together even though they correspond to opposite bias directions and therefore cannot stem from the same transition. We attribute these ambiguities to the aforementioned charge switch.

Finally, we note that some runs terminated in Stage 4 without locating a qubit even though other runs found coherent oscillations at nearly identical plunger coordinates. This behaviour is likely due to sub-optimal barrier voltages.

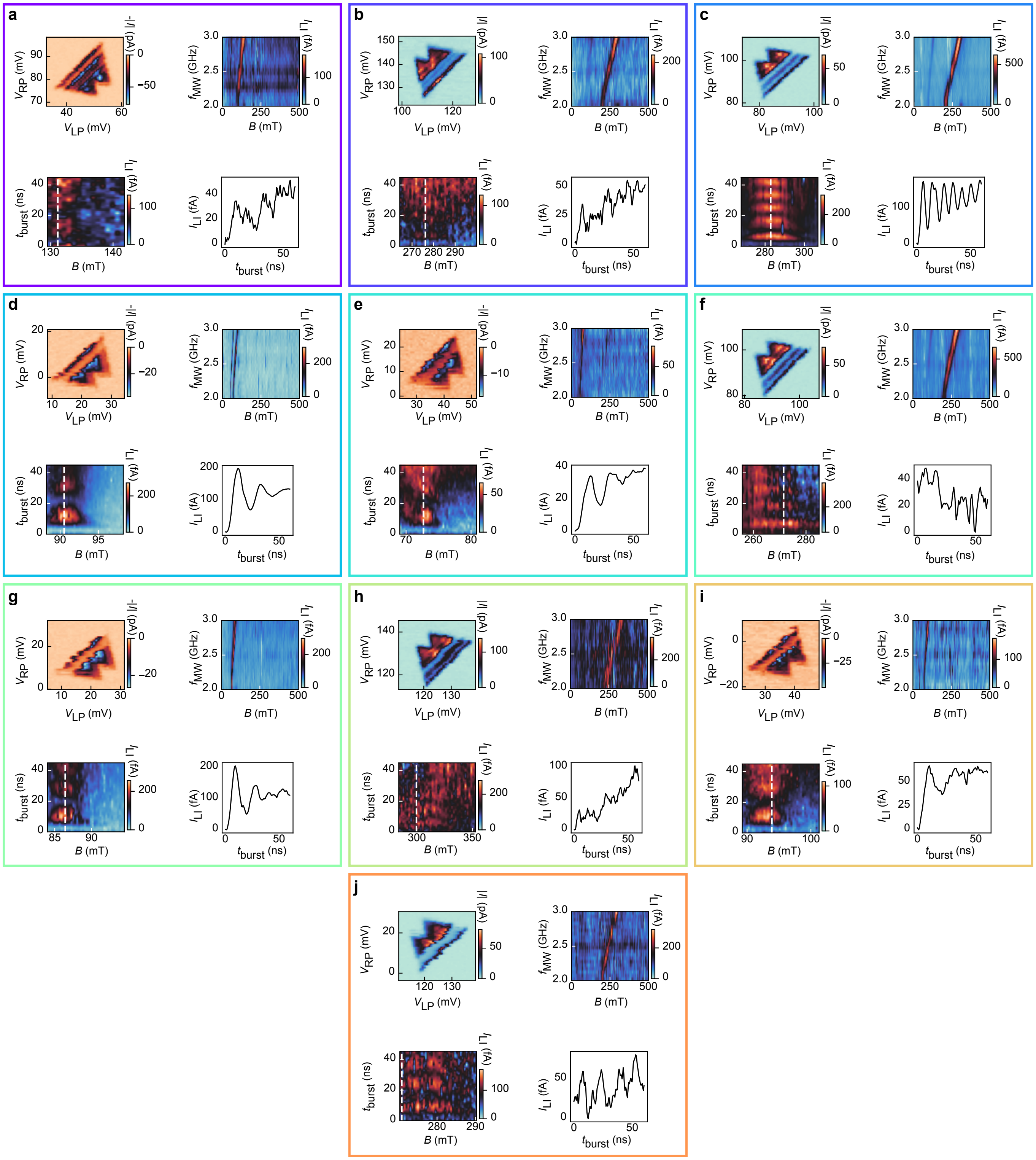

Supplementary Figure 1. **Qubit measurements of all successful runs.** The panels **a,-j,** are ordered by the total run time of the algorithm for each qubit respectively. Each panel includes four current measurements: the pair of bias triangles (upper left), spectroscopy measurement, varying magnetic field and driving frequency (upper right), Rabi chevron pattern, varying magnetic field and burst duration (lower left), and averaged Rabi oscillations (lower right) taken at the dashed lines in the Rabi chevron measurement. All measurements were performed autonomously. The Rabi chevron measurement does not have a dedicated re-centering stage, accounting for the off-centered measurements. The spectroscopy measurements were purely taken for documentation and always with the same ranges; these measurements did not inform any other part of the algorithm. Some measurements for panels **d**, **e**, and **f** were taken again using automated measurements after the initial runs finished because a setting of the lock-in amplifier led to slight measurement artifacts.

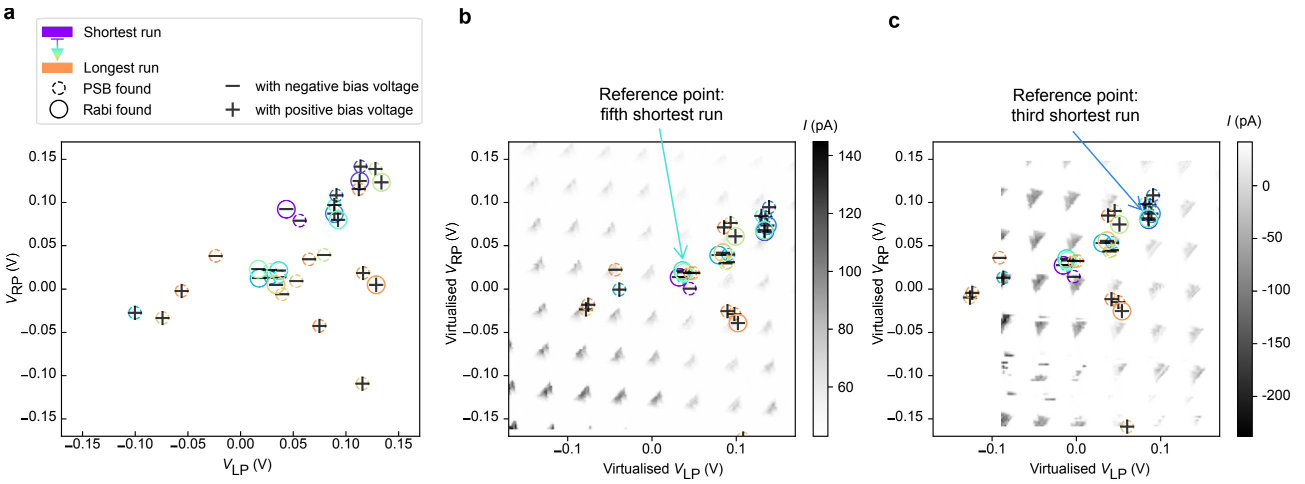

Supplementary Figure 2. **Distribution of PSB and qubit locations in plunger-plunger space. a** Uncompensated plunger voltages. A "+" ("−") indicates that PSB or a qubit was found under positive (negative) bias; symbols share the colour code of Fig. 2b. **b** Plunger coordinates after linear virtual-gate compensation, referenced to the third-shortest successful run; the background shows that run's charge stability diagram. **c** Same data set but compensated with respect to the fifth-shortest run to illustrate the alignment with different charge stability diagrams. Clustering visible in Panels b and c confirms that several runs targeted the same charge transition, while residual mis-alignments highlight the limits of linear compensation in the presence of charge rearrangements.

S3. RELATION OF VISIBILITY TO THE ENERGY-SPLITTING AND ENTROPY SCORES

Spin-state visibility V is a standard figure of merit for qubit readout fidelity, defined as the difference between the measured probabilities of the two spin states, $V = P_{\uparrow} - P_{\downarrow}$ after single-shot thresholding [2, 3]. Direct evaluation of V requires a fully tuned qubit with spin-dependent initialization and single-shot sensing—conditions that are unavailable during the tuning stages. For real-time, closed-loop tuning we therefore employ two surrogate metrics that are accessible from routine DC measurements yet correlate with visibility:

Energy-splitting score (Fig. 2b-ii) The ratio of current in the "valley" next to the bias-triangle baseline to the average current in the excited-state region. A deeper dip indicates a larger singlet–triplet gap Δ_{ST}, which energetically separates the relevant singlet and triplet states from leakage states and thereby improves the maximum achievable visibility.

Entropy score (Fig. 2d-ii) The Shannon entropy of the current trace recorded while sweeping magnetic field at a fixed pulse condition. A well-defined resonance peak lowers the entropy, signalling strong spin-contrast.

Consequently, a higher energy-splitting score and lower entropy both promote large visibility, but, crucially, they can be computed before full qubit calibration. This makes them ideal objective functions for autonomous tuning. The scores thus act as practical, physics-informed proxies for visibility throughout the automated workflow.

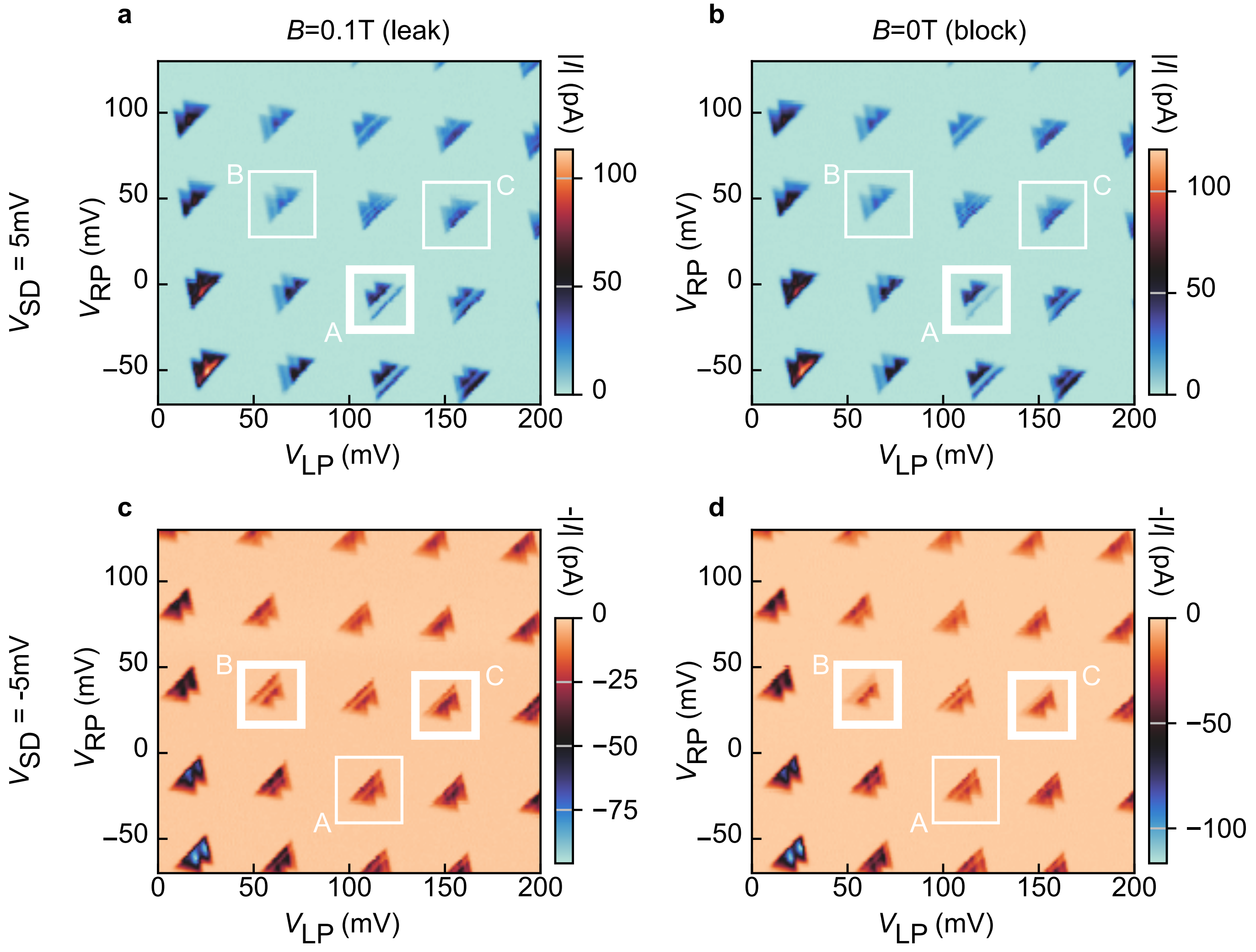

Supplementary Figure 3. **Charge transitions investigated.** There are three charge transitions that we consider in the following, **A**-**C**. There are four measurements of each transition, with positive (**a, b**) and negative bias (**c, d**) voltage, and without a magnetic field (**b, d**) and with a magnetic field at $B = 0.1$T (**a, c**), which should lift PSB. Transition **A** shows PSB with a positive bias voltage. Transitions **B** and **C** show PSB with a negative bias voltage.

S4. EMPIRICAL JUSTIFICATION FOR THE ENERGY-SPLITTING SCORE

The energy-splitting score was grounded in experimental experience. Given the tools developed here, we can gather the data necessary to provide justification.

We study three different charge transitions, labeled **A** through **C**, as shown in Supplementary Fig. 3. Transition **A** show PSB in positive bias direction, while transitions **B** and **C** display it in the negative bias direction.

Measurements of the detuning current traces, both with and without an applied magnetic field, enable the comparison of currents of bias triangles where readout was successful with currents where readout failed. For this analysis, we employed a lenient criterion to define successful readout: the presence of a discernible EDSR peak during the magnetic field sweep. Supplementary Fig. 4 displays these detuning current traces for transitions **A-C**, annotated with mean and median values.

The current within the valley is particularly interesting. Consider the top two panels for each transition in Supplementary Fig. 4. There is a “valley” of the current which we mark with a green star. The concept is also introduced in Methods (Stages of the algorithm, Stage 2: Tune barriers, a. Barrier optimisation).

Those currents in the valley are extracted and visualised in the first row of Supplementary Fig. 5. To preserve the visual interpretability of these plots, we clip the current in valley plots at 50 pA as currents for transition **B** can far exceed this value for some cases. Comparing the current at this point for the cases of failed and successful readout indicates that lower currents generally correlate with successful readout. Overlaps in the data distributions suggest that these measurements alone are not definitive indicators of readout success.

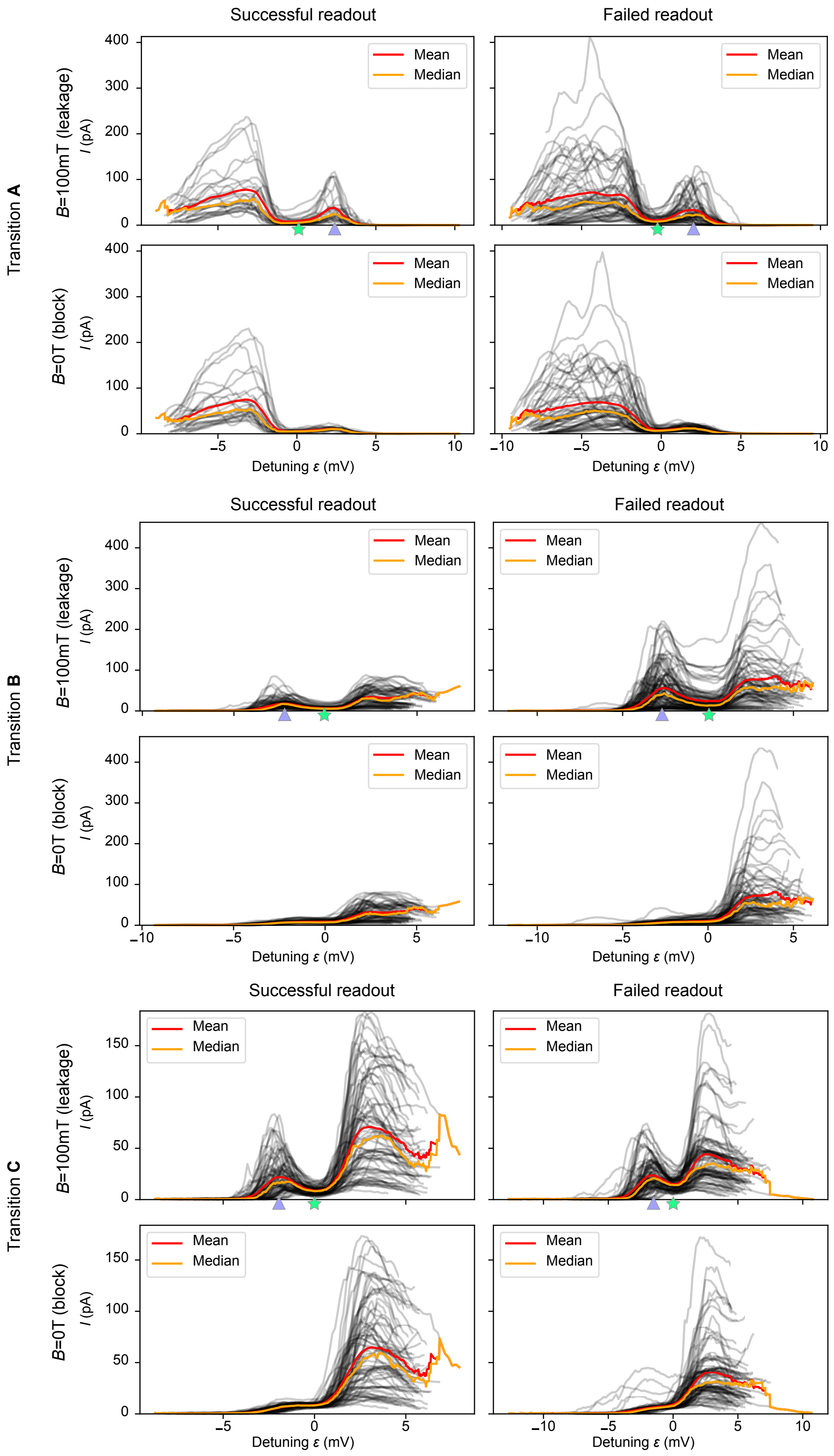

Supplementary Figure 4. **Detuning line measurements.** For transitions **A-C**, we show measurements of current along the detuning line for varying barrier voltages for four different conditions: with and without a magnetic field, and with successful and failed readout, as determined by manual labels. The traces are centered around the identified valley point, which is put to be at 0 detuning. This is unconventional because the energy levels align at the base line which usually would be identified with 0 detuning. This choice allows for improved visual clarity. The green stars and purple triangles indicate the positions at which the current is taken for further analysis, see Supplementary Fig. 5.

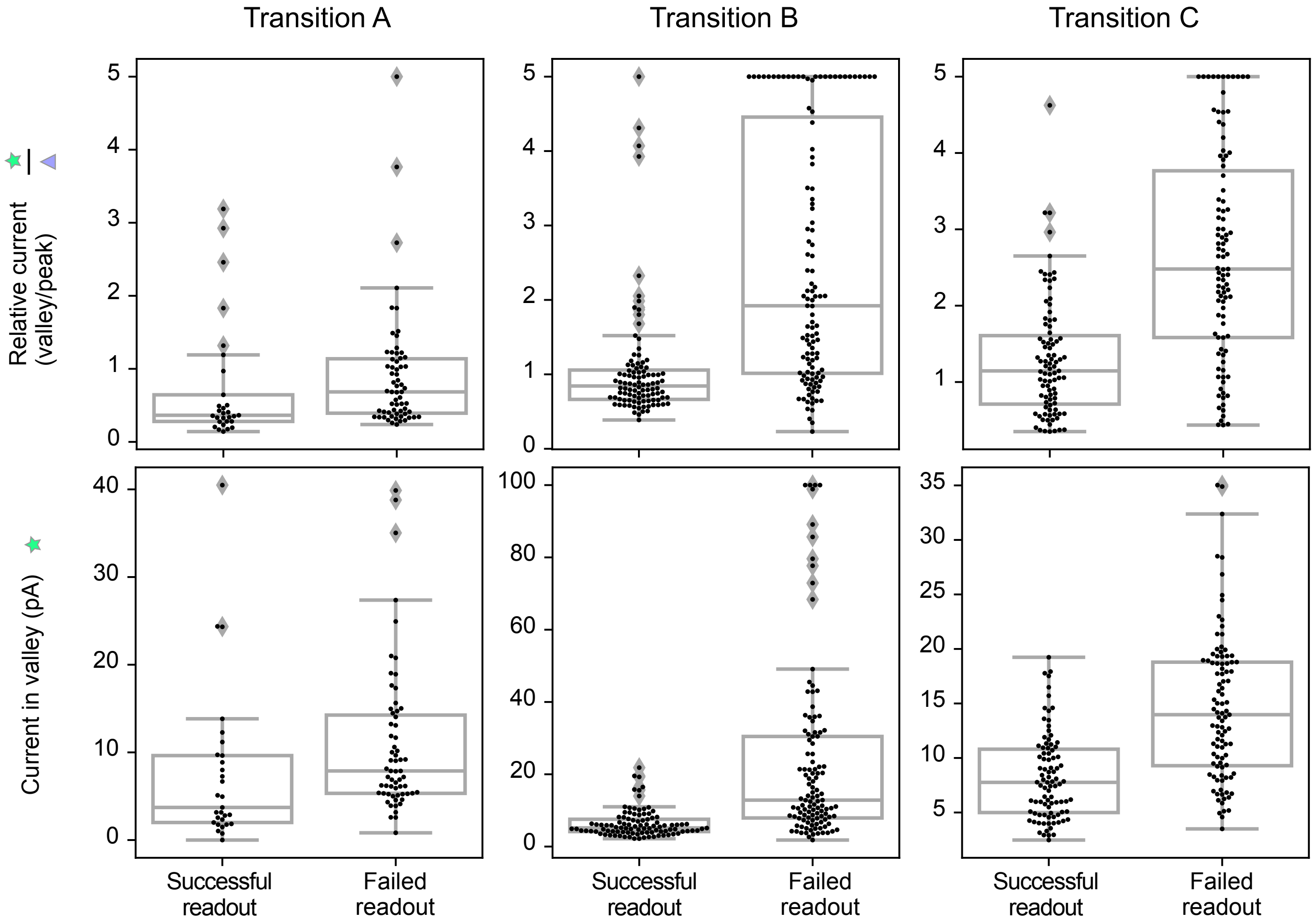

Supplementary Figure 5. **Comparisons of the currents on the detuning line.** The underlying data from Supplementary Fig. 4 is analysed further by only considering certain currents or current ratios. We show the individual data as points and the statistics as underlying box plots. The two metrics under consideration are the ratio of currents in the valley and the current at the base line at high magnetic field (top row), the current in the valley (bottom row). The data for the valley current is clipped at 50 pA for visual clarity. For transition **A**, there are 60 data points with failed readout, 27 with successul readout, for transition **B**, there are 94 with failed readout, 81 with successful readout, for transition **C**, there are 112 with failed readout, 98 with successful readout.

In the context of tuning, just aiming for lower currents might be unwise because that would lead us into a fully pinched regime. We can normalise the current in the valley by dividing it by the current at the baseline, marked by a purple triangle in Supplementary Fig. 4. The resulting ratio is shown in the top row of Supplementary Fig. 5. We see a similar effect, as when just considering the current in the valley, although especially for transition **B** the predictive insight seems weakened. Still, this result provides justification for the choice of the score function made in Stage 2.

Despite the insights gained, the signals derived from these measurements are not perfect predictors of readout success. Variabilities such as inaccurately chosen g-factors, suboptimal pulse durations, or misaligned readout points can significantly affect outcomes.

S5. HYPERPARAMETERS

List of all hyperparameters and comment on what they do. We do not report on hyperparameters that are irrelevant to the functionality of the algorithm, such as how often measurements are plotted to a documentation file.

1. Define DQD

a. Hypersurface building

- Steering parameters:
  - `number_of_rays` = 32 // Number of rays used to build hypersurface.
  - `n_noise_floor` = 100 // Number of measurements of the noise floor.
- Measurement parameters:
  - `lower_bounds` = $[0, 0, 0]$ // Defines lower bound of area in which the hypersurface model is built, in V.
  - `upper_bounds` = $[1.8, 1.8, 1.8]$ // Defines upper bound of area in which the hypersurface model is built, in V.
  - `bias_low` = 0.0007 // Low bias voltage used to find pinch off starting from the conducting region, in V.
  - `bias_high` = 0.005 // Bias voltage used to in subsequent stages. Used to confirm pinch off going from non-conducting to conducting region, in V.
  - `d_r` = 0.003 // Step length of pinch off search, in V.
  - `len_after_pinchoff` = 0.250 // Length after last point above threshold before pinch off is considered true (past last Coulomb peaks), in V.
  - `max_dist` = 2 // Additional safe range how far the ray is maximally ramped, in V.
- Analysis parameters:
  - `threshold_as_multiple_of_noise_high` = 100 // Used to define the noise threshold based on the noise floor measurement

b. Double dot detection

- Measurement parameters:
  - `magnetic_field` = 0.1 // At which magnetic field the measurements are taken, in T.
  - `plunger_location` = $[0, 0]$ // Center of the plunger gate voltages, in V.
  - One-dimensional scan (to detect Coulomb peaks):
    - `window_right_plunger` = 0.1 // Defines side length of window in which measurements us taken, in V.
    - `window_left_plunger` = 0.1 // Defines side length of window in which measurements us taken, in V.
    - `n_px` = 128 // Number of points to be taken.
    - `wait_time` = 0.051 // Delay after setting parameter before measurement is performed, in s.
  - Two-dimensional scan (to detect DQD features):
    - `window_right_plunger` = 0.2 // As above.
    - `window_left_plunger` = 0.2 // As above.
    - `n_px_rp` = 48 // Number of points to be taken for right plunger axis.
    - `n_px_lp` = 48 // Number of points to be taken for left plunger axis.
    - `wait_time_slow_axis` = 0.5 // Delay after setting parameter before measurement is performed, in s.
    - `wait_time_fast_axis` = 0.051 // Delay after setting parameter before measurement is performed, in s.
- Analysis parameters:
  - `max_distance_between_locations` = 0.1 // Used to determine number of points sampled within DQD search region. Sets maximal distance between each sampled point, in V.
  - `path_to_nn` = `local_path` // Path to weights of neural network used for DQD feature detection.

2. Tune barriers

a. Entropy optimisation

- Steering parameters:
  - seeding = 15 // Parameter for Bayesian optimisation that informs exploration period.
  - n_required_results = 30 // Number of stability diagrams to be taken.
- Measurement parameters:
  - rp_start = −0.15 // Starting point of measurement for right plunger, in V.
  - rp_end = 0.15 // End point of measurement for right plunger, in V.
  - lp_start = −0.15 // Starting point of measurement for left plunger, in V.
  - lp_end = 0.15 // End point of measurement for left plunger, in V.
  - n_points_plungers = 100 // Number of points in each dimension.

b. Plunger window detection

- Steering parameters:
  - number_of_full_scans_threshold = 10 // Maximum number of full scans (i.e., without the efficient measurement algorithm) to be taken.
  - number_of_candidates = 10 // Maximum number of candidates Stage 2 can suggest in each bias direction, i.e., 10 can lead to up 20 candidates.
  - bias_directions = [positive_bias, negative_bias] // Candidates are built in those bias directions.

3. Find PSB

a. Wide shot PSB detection

- Steering parameters:
  - max_number_candidates = 5 // Maximum number of candidates this sub-stage can create.
- Measurement parameters:
  - low_magnetic_field= 0.0 // Magnetic field at which the stability diagram with blocked current shall be taken, in T.
  - high_magnetic_field= 0.1 // Magnetic field at which the stability diagram with leakage current shall be taken, in T.
  - resolution = 0.002 // Resolution of stability diagram in each axis, in V.
  - padding = 0.03 // Padding added to the plunger window suggestion from previous stage. Needed to have a slight margin around bias triangles, in V.
- Analysis parameters:
  - psb_threshold = 0.5 // Threshold for PSB detection. Neural network returns a value between 0 and 1 for each pair of bias triangles.
  - folder_path_to_nn = local_path // Path to neural network model that predicts signatures of PSB from low resolution measurements.
  - offset_px = 10 // Parameter used in the location detection via auto-correlation. The highest peak will always be in the center, so peaks within a certain distance (given in pixel here) from the center are disregarded.

b. Re-centering

- Measurement parameters:
  - `magnetic_field`= 0.0 // Magnetic field at which the stability diagram shall be taken, in T.
  - `resolution` = 0.002 // Resolution of stability diagram in each axis, in V.
  - `wait_time_slow_axis` = 0.5 // As above.
  - `wait_time_fast_axis` = 0.051 // As above.
- Analysis parameters (all related to routine from Kotzagiannidis *et al.* [4]):
  - `segmentation_upscaling_res` = 2 // Image is upscaled by this factor to improve segmentation.
  - `relative_min_area` = 0.01 // Computes the `min_area` as a fraction of the total area. `min_area` sets a threshold for the minimum area of contour to be detected to avoid outliers.
  - `denoising` = true // Apply Gaussian smoothing.
  - `allow_MET` = false // Determines whether the 'Minimal enclosing triangle' technique is used or not. If true facilitates enclosing triangle shape approximation for disconnected contours.
  - `thr_method` = 'triangle' // Thresholding method for contour detection.

c. High resolution PSB detection

- Measurement parameters:
  - `low_magnetic_field`= 0.0 // As above.
  - `high_magnetic_field`= 0.1 // As above.
  - `resolution` = 0.00075 // As above.
  - `wait_time_slow_axis` = 0.5 // As above.
  - `wait_time_fast_axis` = 0.051 // As above.
  - `padding` = 0.005 // As above.
- Analysis parameters:
  - `slope_tol` = 0.4 // Tolerance for deviation in absolute value between slopes of detected lines.
  - `int_tol` = 0.05 // Tolerance for PSB metric (absolute value difference between normalized segment intensities).
  - `seg_tol` = 0.05 // Gives percentage of image length as threshold for segments that are too small.
  - `median` = false // If true, selects the median of detected lines (ordered by y-intercept); false by default, so that the line with largest y-intercept (outmost) is selected.
  - `segmentation_upscaling_res` = 2 // As above.
  - `relative_min_area` = 0.01 // As above.
  - `denoising` = true // As above.
  - `allow_MET` = false // As above.
  - `thr_method` = 'triangle' // As above.

d. Danon gap check

- Measurement parameters:
  - `magnetic_field_min` = - 0.1 // Start of magnetic field, in T.
  - `magnetic_field_max` = 0.1 // End of magnetic field, in T.
  - `resolution_magnet` = 0.003 // Resolution of magnetic field, in T.

- resolution_detuning = 0.0001 // Resolution of detuning line axis, in V.
- detuning_base_offset = 0.002 // We add this to the detuning line measurement to include the full base as the segmentation algorithm can lead to detuning line definitions that end on the base line, therefore missing valuable information.
- extra_wait_time_slow_axis = 0.5 // The slow axis (magnetic field) is delayed by the time needed for the magnet to ramp one position, plus this given time.
- wait_time_fast_axis = 0.077 // As above.

• Analysis parameters:

- segmentation_upscaling_res =2 // As above.
- min_area = 3 // As above.
- thr_method = 'triangle' // As above.
- allow_MET = false // As above.
- padding_factor = 1 // As above.
- minimum_det_line_length_ratio = 0.33 // The detuning line is determined via the segmentation algorithm. It also determines a cutoff within the triangles so that the algorithm only takes measurements at the base line of the triangle. If the detuning line that is determined is less than minimum_det_line_length_ratio of the full detuning line (from base line to the tip of the triangles), we extend the detuning line definition to avoid detuning lines definitions that are too short.
- peak_offset_tolerance = 0.025 // The gap can be at most offset from 0T by this much and still be accepted as a true gap, in T.
- sigma = 1 // For the gap detection, Gaussian smoothing factor.
- field_gap_size = 0.002 // For the gap detection, parameter that controls maximal gap width.
- relative_depth = 1.0 // For the gap detection, parameter that controls maximal gap depth.

4. Find readout

a. Entropy optimisation

• Steering parameters:

- number_of_candidates = 3 // Maximum number of candidates this sub-stage can create.
- seeding = 15 // As above.
- iterations = 30 // Number of total measurements taken by the Bayesian optimisation.
- freq_vs.minimum = 2.6e9 // Minimum driving frequency f_{MW} used in Bayesian optimisation, in Hz.
- freq_vs.maximum = 2.9e9 // Maximum driving frequency f_{MW} used in Bayesian optimisation, in Hz.
- burst_time_ns.minimum = 2 // Minimum burst time t_{burst} used in Bayesian optimisation, in ns.
- burst_time_ns.maximum = 16 // Minimum burst time t_{burst} used in Bayesian optimisation, in ns.

• Measurement parameters:

- magnetic_field = 0.1 // As above.
- resolution = 0.00075 // As above.
- padding = 0.005 // As above.
- wait_time_slow_axis = 0.5 // As above.
- wait_time_fast_axis = 0.051 // As above.
- lockin_tc = 1 // Time constant of lock-in amplifier, in s.
- field_setpoint.start = 0.0 // Minimum magnetic field, in T.
- field_setpoint.stop = 0.4 // Maximum magnetic field, in T.

  - `field_setpoint.num_points` = 300 // Number of points in magnetic field axis.
  - `field_setpoint.delay` = 1 // Delay parameter, as above.
- Analysis parameters:
  - `segmentation_upscaling_res` = 2 // As above.
  - `relative_min_area` = 0.001 // As above.
  - `thr_method` = 'triangle' // As above.

b. Resonance confirmation

- Measurement parameters:
  - `magnetic_field_window` = 0.1 // Window symmetric around the assumed peak, in T.
  - `resolution_magnet` = 0.001 // Resolution of scan, in T.
  - `wait_time` = 2.5 // Delay for measurement, needs to be longer than the lock-in time constant.
  - `lockin_tc` = 2 // As above.
- Analysis parameters:
  - `prominence` = 0.9 // Minimum prominence of peaks.
  - `sigma` = 1 // Gaussian smoothing factor.
  - `peak_offset_tolerance` = 0.025 // Peaks with a maximum offset of this parameter from the assumed position are accepted.

c. Spectroscopy

- Measurement parameters:
  - `min_magnetic_field` = 0 // Start of magnetic field, in T.
  - `max_magnetic_field` = 0.5 // End of magnetic field, in T.
  - `resolution_magnet` = 0.005 // Resolution of magnetic field, in T.
  - `min_freq_vs` = 2e9 // Start of driving frequency f_{MW}, in Hz.
  - `max_freq_vs` = 3e9 // End of driving frequency f_{MW}, in Hz.
  - `resolution_freq` = 0.5e8 // Resolution of driving frequency f_{MW}, in Hz.
  - `extra_wait_time_slow_axis` = 1 // As above.
  - `wait_time_fast_axis` = 1.1 // As above.
  - `lockin_tc` = 1 // As above.

d. Rabi chevron

- Measurement parameters:
  - `magnetic_field_window_multiplier` = 2 // The window is determined by the width of the peak that resonance confirmation (Stage 4b) takes and multiplied with this factor.
  - `n_px_magnet` = 40 // Number of points in the magnetic field axis.
  - `resolution_burst_time` = 1e-9 // Resolution of the burst time t_{burst} axis, in s.
  - `min_burst_time` = 0 // Minimum of the burst time t_{burst}, in s.
  - `max_burst_time` = 45e-9 // Maximum of the burst time t_{burst}, in s.
  - `dead_burst_time` = 10e-9 // The total length of the pulse is twice the maximum t_{burst}, plus this factor, in s.
  - `extra_wait_time_slow_axis` = 6 // As above. Needs to be significantly larger than the lock-in time constant to avoid spill-over effects.
  - `wait_time_fast_axis` = 2.5 // As above.
  - `lockin_tc` = 2 // As above.

e. Rabi oscillations

- Steering parameters:
  - `n_repetitions` = 5 // Number of repetitions of the same Rabi oscillation measurement.
- Measurement parameters:
  - `resolution_burst_time` = 0.5e-9 // As above.
  - `min_burst_time` = 0 // As above.
  - `max_burst_time` = 60e-9 // As above.
  - `dead_burst_time` = 10e-9 // As above.
  - `lockin_tc` = 2 // As above.

S6. EFFICIENT MEASUREMENT ALGORITHM

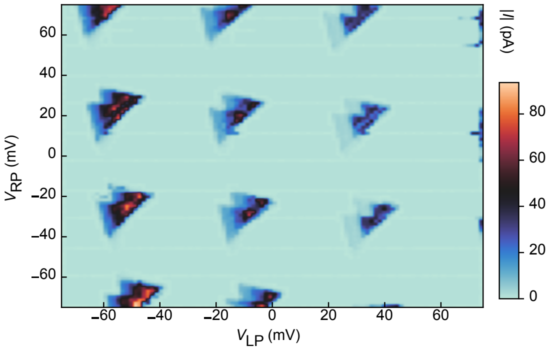

Supplementary Figure 6. **Examples of measurements taken with the efficient measurement algorithm.** Areas where no measurement have been taken have been filled with the threshold value.

Taking multiple charge transition stability diagram measurements is notably time-consuming, largely due to the predominance of featureless areas, as bias triangles are typically embedded within a skewed rectangular pattern. To address this, we have developed an efficient measurement algorithm. By rephrasing the measurement of bias triangles as an image processing task, whereby the goal is to determine a contour that traces the outline of a bias triangle, we were able to reduce the measurement time to 33% of a brute force scan.

For a binary image represented by a matrix composed entirely of zeros and ones, the perimeter of any grouping of non-zero elements that form a contiguous region is known as a *contour*. The Moore-Neighbour contour tracing algorithm provides a method of evaluating a complete contour given a starting point within the contour [5]. The Moore-Neighbour contour achieves this by only ever examining pixels adjacent to a previously examined pixel. As the location of pixels corresponds to plunger gate voltages, measurements of well separated pixels are both costly in time and present a risk of introducing noise such as switches. Once the edge of a bias triangle has been identified, its contour can therefore be quickly measured with minimal overhead from the device. After the contour has been evaluated, each pixel inside the contour can be measured sequentially to complete the bias triangle.

To construct a binary image from a series of measured current values at differing gate voltages, a threshold must be determined. Current values above and below this threshold are considered to be ones and zeros in the binary image, respectively. The threshold can either be set manually, using prior knowledge of the system, or determined on-the-fly. To provide a fully automated system we took a calibration scan using a sparse sampling and evaluated the median absolute deviation threshold from these measured points. The sampling routine was a so-called *snake scan*, where measurements are performed horizontally left-to-right until a boundary of the measurement region is reached, then proceed vertically for a fixed length and continue horizontally in the opposite direction until the entire image has been covered.

With a threshold determined, a second sparse sampling across the measurement region is performed. The Moore-Neighbour contour tracing routine is triggered on any measurements that exceed the threshold, followed by a routine to measure the inside of the contour. This process systematically captures a complete pair of bias triangles. Post completion, the scan resumes until the next cluster necessitates flood fi lling. In order to minimize wasted measurements, current values were ca ched. The second sampling of the routine also used a snake scan to explore the measurement region, however it was offset vertically compared to the original to maximise the chance of encountering a bias triangle.

Bias triangles are organised on a skewed grid pattern. The bias triangles evaluated in the second sparse sampling stage can be used to fit a skewed rectangular grid and infer the location of any missing bias t riangles. Triangles can be missed by the initial sparse sampling if they reside between the horizontal lines of the two snake scans. A skewed grid can be represented by two vectors that describe the spatial separation between points on the grid, and the location of one grid point. These parameters were determined by minimising the total distance between the barycenter of all contours evaluated in the second sampling stage and points on the fitted grid. After fitting, each point on the grid within the measurement region that did not have a bias triangle was measured sequentially.

Employing this method has proven to significantly streamline the process, cutting down the measurement time by approximately two-thirds. For the hyperparameters as reported above, the measurement of a 100 by 100 point stability diagram takes 14.5 min ± 3.0 min with this efficient measurement algorithm, and 43.7 min ± 0.1 min with a conventional grid scan.

S7. SEARCH TREE EXAMPLES

In Fig. 1b of the main text, we show an illustrative example of a search tree. Here, we show the search trees that were actually constructed for the longest and shortest runs in our experiments.

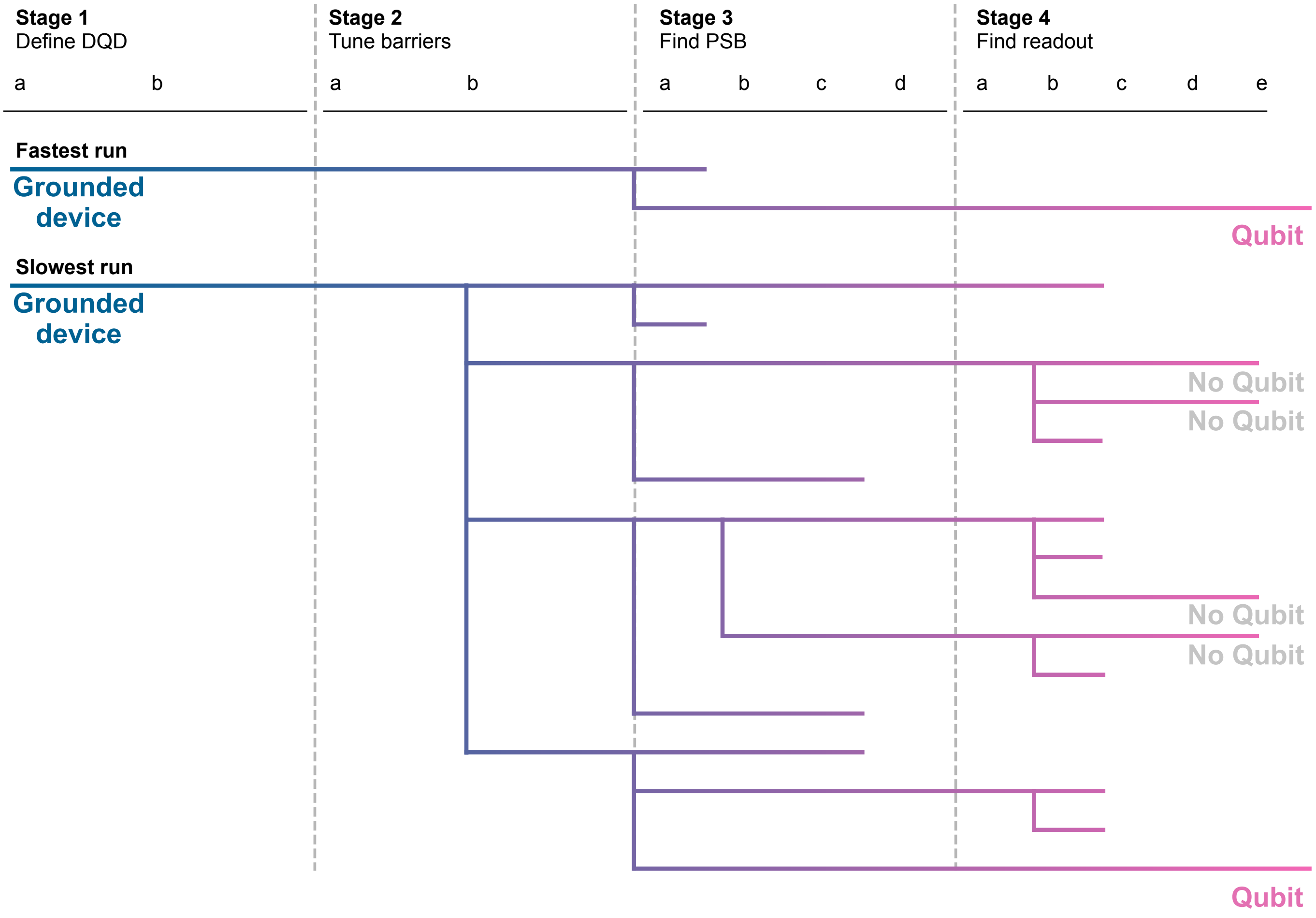

Supplementary Figure 7. **Examples of search trees from full runs.** The fastest run only has two branches and then successfully found a qubit. The slowest run explored much more, with several branches reaching all the way to qubit measurements. However, only the last branch shows conclusive qubit signatures. We rejected the first tries as noise.

S8. MODULAR FRAMEWORK

We implemented several design choices to standardize the framework across all stages, achieving a cohesive and modular architecture. Each stage exhibits these common characteristics:

1. Stage structure

1. Integration with QCoDeS [6]: All stages have access to the station object of QCoDeS, allowing each stage to take measurements and change experimental parameters.
2. Data management: A data access object manages (in addition to the QCoDeS database) costum data saving, such as the structure of the tree that was created so far, and automated documentation of the run.
3. Hyperparameter configuration: Each stage possesses specifically tailored hyper-parameters to fulfill its requirements, as detailed in the Section S5.
4. Candidate management: A list of candidates that were passed to a stage and that are sent off to another stage is kept.

a. Functions

1. Investigation function: Stages are primarily invoked through an `investigate` function, managing candidate lists, orchestrating measurements and data analysis, and forwarding candidates to the subsequent stage.
2. Experimental setup: A `prepare_experiment` function sets up the experimental parameters as needed, for example, setting certain voltages, ramping the magnet to a starting position, or stopping the AWG from outputting a pulse sequence.
3. Experiment execution: The function `perform_experiment` unction conducts the stage-specific measurements.
4. Data analysis: `determine_candidate` function analyses the acquired data to assemble a viable set of candidates for further exploration.

2. Candidates

1. Data association: Once a stage has taken data relating to a specific candidate, it will keep a note of the global unique identifier (GUID) that is recorded in the QCoDeS database.
2. Parameter storage: Critical parameter information is stored flexibly in a dictionary format to adapt to various experimental scenarios.
3. Metadata storage: Candidates carry metadata, such as their position within the search tree.
4. Stage timing: The duration required for each stage's process is recorded.
5. Resulting candidates list: A distinct list is maintained for candidates resulting from the stage's analysis.

[1] A. C. Johnson, J. R. Petta, C. Marcus, M. Hanson, and A. Gossard, Physical Review B **72**, 165308 (2005).
[2] J. Z. Blumoff, A. S. Pan, T. E. Keating, R. W. Andrews, D. W. Barnes, T. L. Brecht, E. T. Croke, L. E. Euliss, J. A. Fast, C. A. Jackson, et al., PRX Quantum **3**, 010352 (2022).
[3] D. Keith, S. Gorman, L. Kranz, Y. He, J. Keizer, M. Broome, and M. Simmons, New Journal of Physics **21**, 063011 (2019).
[4] M. Kotzagiannidis, J. Schuff, and N. Korda, arXiv preprint arXiv:2312.03110 (2023).
[5] T. Pavlidis, Algorithms for graphics and image processing (Springer Science & Business Media, 2012).
[6] Copenhagen / Delft / Sydney / Microsoft quantum computing consortium, "QCoDeS," .
